# Genome Wide Association for Addiction: Replicated Results and Comparisons of Two Analytic Approaches

**DOI:** 10.1371/journal.pone.0008832

**Published:** 2010-01-21

**Authors:** Tomas Drgon, Ping-Wu Zhang, Catherine Johnson, Donna Walther, Judith Hess, Michelle Nino, George R. Uhl

**Affiliations:** Molecular Neurobiology Branch, National Institutes of Health Intramural Research Program, National Institute on Drug Abuse, Baltimore, Maryland, United States of America; University of Muenster, Germany

## Abstract

**Background:**

Vulnerabilities to dependence on addictive substances are substantially heritable complex disorders whose underlying genetic architecture is likely to be polygenic, with modest contributions from variants in many individual genes. “Nontemplate” genome wide association (GWA) approaches can identity groups of chromosomal regions and genes that, taken together, are much more likely to contain allelic variants that alter vulnerability to substance dependence than expected by chance.

**Methodology/Principal Findings:**

We report pooled “nontemplate” genome-wide association studies of two independent samples of substance dependent *vs* control research volunteers (n = 1620), one European-American and the other African-American using 1 million SNP (single nucleotide polymorphism) Affymetrix genotyping arrays. We assess convergence between results from these two samples using two related methods that seek clustering of nominally-positive results and assess significance levels with Monte Carlo and permutation approaches. Both “converge then cluster” and “cluster then converge” analyses document convergence between the results obtained from these two independent datasets in ways that are virtually never found by chance. The genes identified in this fashion are also identified by individually-genotyped dbGAP data that compare allele frequencies in cocaine dependent *vs* control individuals.

**Conclusions/Significance:**

These overlapping results identify small chromosomal regions that are also identified by genome wide data from studies of other relevant samples to extents much greater than chance. These chromosomal regions contain more genes related to “cell adhesion” processes than expected by chance. They also contain a number of genes that encode potential targets for anti-addiction pharmacotherapeutics. “Nontemplate” GWA approaches that seek chromosomal regions in which nominally-positive associations are found in multiple independent samples are likely to complement classical, “template” GWA approaches in which “genome wide” levels of significance are sought for SNP data from single case *vs* control comparisons.

## Introduction

Vulnerability to addictions is a complex trait with substantial genetic influences that are documented by data from family, adoption and twin studies [1]–[4]. Twin studies also document shared heritable influences on vulnerability to dependence on addictive substances from different pharmacological classes (*eg* nicotine and stimulants) [2], [3], [5]. In individuals from most populations, each gene's variants are likely to contribute modestly to substance dependence vulnerability. Contributions of nicotinic receptor gene variants to individual differences in smoking quantity [6]–[10] and acetaldehyde dehydrogenase/alcohol dehydrogenase variants to risk for alcohol dependence in Asians may provide larger effects of variants at single loci [11], [12]. However, combined data from linkage and initial genome wide association studies (GWA) [6], [13]–[17] suggest that most genetic effects on vulnerability to substance dependence are likely to be polygenic.

GWA is a method of choice for identifying genes whose variants influence vulnerability to complex disorders. GWA approaches that we term “template” seek to identify “genome wide” levels of significance (*ca* 10^−7^–10^−8^) for case *vs* control differences in single samples of individually genotyped individuals. “Replication” of GWA results in these “template” GWA analyses is based on identification of genome wide significance for the same SNP with the same phase of association in each of multiple independent samples.

However, with underlying polygenic genetic architectures, effects of only modest magnitude are likely to be identified in many single samples of practical size. We and others have developed “nontemplate” GWA analyses to address highly heritable complex phenotypes for which there is little evidence for many genes of major effect. These analyses have focused on identification of nominally significant case *vs* control allele frequency differences at several nearby SNP markers in multiple independent samples. Identifying “clustered” positive findings at several nearby SNPs and finding clustered positive results in several independent samples provide some of the best available controls for technical errors and for the large numbers of repeated comparisons that are fundamental to GWA. There is no consensus concerning criteria for declaring “replication” of GWA results in the absence of genome wide significance for the same SNP with the same phase of association in multiple independent samples [18]–[23]. Several considerations have prompted differing approaches to 1) combining and comparing GWA datasets and 2) declaring that association between sets of nearby SNPs and a complex disorder is “replicated” in the absence of genome wide significance for any result. Underlying functional haplotypes contributing to disease vulnerability may be tagged differently by different SNP sets in different samples. Allelic heterogeneity can result in 1) contributions of different variants within the same gene and 2) differences in the predominant variants in a gene that influence the phenotype in different samples. Meta-analyses often combine data from studies that have examined alleles of different sets of SNPs.

We now report “nontemplate” GWA [24] studies that compare allele frequencies for almost 870,000 autosomal SNPs in each of two independent samples (one European-American and the second African-American) of controls *vs* polysubstance abusers who report heavy use and dependence on at least one illegal substance. We have characterized and collected these case and control samples at a single site. We use a nontemplate GWA approach with DNA pooling to study the genetics of this illegal behavior. We analyze genes that are identified by “replicated” results from these data in each of two ways that appear to complement each other (though they are not independent of each other): 1) “converge then cluster”, based on identification of SNPs within a gene that a) display nominally significant case *vs* control allele frequency differences in each of these two samples and b) lie near other SNPs with the same properties, and 2) “cluster then converge”, based on SNPs within genes that are identified in each of the two samples by clusters of SNPs that a) display nominally significant case *vs* control allele frequency differences and b) lie near other SNPs with the same properties. Since approach (2) does not require that the identical SNPs display nominally significant results in each of several samples, it is especially useful for evaluating concordance between GWA datasets that use different sets of SNPs. We can thus apply this approach to examining the concordance between addiction and co-occurring traits likely to display complex genetic influences [25] using empirical Monte Carlo statistics to assess the significance of results. We discuss this work in light of its technical and analytic limitations and in its similarities and differences with “template” GWA analyses that seek associations that display genome-wide significance, typically in phenotypes that display oligogenic genetic architectures and/or in larger samples that are often recruited in multiple locations. We also describe the ways in which these pooled genotype data identify a number of the same genomic regions that are identified by recently available dbGAP datasets that provide individual genotyping for cocaine-dependent and nondependent comparison groups.

## Results

### Variation and Power Calculations

**Figure 1:**
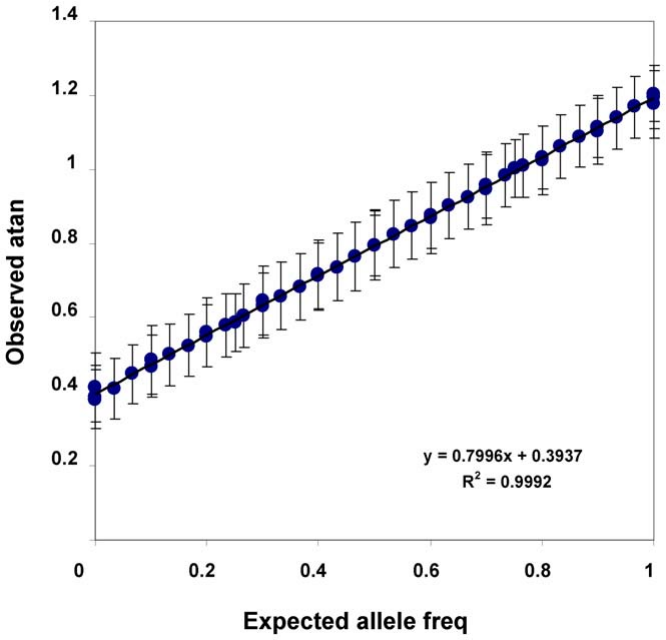
Validation graph of the relationships between observed (y axis) and expected (x axis) allele frequency data for Affymetrix 6.0 arrays. “Expected” frequencies come from individual genotyping of individuals. These individuals were assigned to three sets of pools each containing 2, 5 and 15 CEPH individuals (total of 81 individuals). Arctan A/B represent the “observed” measures of allele frequency and are arctangents of the A/B hybridization ratios for this set of pools of individuals. In this figure we have only used SNPs that show at least 10% difference in the expected values across the set of pools (total of 146,000 SNPs). We have obtained similar data from studies validating 500k,100k, 10k and HuSNP arrays [14]–[16]. Note that DNA used for hybridization is less than that recommended for individual genotyping (135 *vs* 225 ng) in order to avoid saturation of hybridization signals for some array features. Error bars indicate SEM.

The pooling approach used herein provides evidence for good assessment of allele frequency differences and variation in these estimates. SNP allele frequency assessments made herein display modest variability and good fits between individual and pooled genotyping with mean correlation of 0.98+/−0.002 (standard error, SEM, Fig. 1). Validating studies with similar arrays add to confidence in this data [13], [14], [26]–[34]. SEM for the variation among three replicate studies of each DNA pool was +/−0.03. SEM for the variation between the *ca*. 20 pools studied for each ethnicity/phenotype group was +/−0.02. These estimates of variability allowed us to estimate 0.8 and 0.9 power to detect 5 and 10% allele frequency differences in the African American sample sizes described here. We had 0.76 and 0.99 power to detect 5 and 10% allele frequency differences in European American samples. Corresponding false negative probabilities for approach 1 (*converge then cluster*) are thus 0.39 and 0.11, since this approach requires nominally positive results for the same SNP from both samples. Statistical power for the analysis of these samples can also be calculated using “gene detective” [25]; this power rises from 0.1 to *ca.* 0.9 as risk to a sib of an affected individual (λ_s_) rises from 1.2 to 2.5 [35].

### “Converge Then Cluster”

In our assessment of allele frequency differences between abusers and controls 83,202 and 75,327 SNPs displayed “nominally positive” t values with p<0.05 in African- and European-American samples, respectively. There was substantial convergence of the results from these two GWA datasets using the non-template (1) “converge then cluster” GWA analysis approach. 11,037 of the 870,000 tested SNPs displayed “reproducible” results, as defined using this approach. These SNPs thus displayed nominally significant abuser *vs* control allele frequency differences in each of the two samples. This overall convergence was much greater than anticipated based on chance. None of 100,000 Monte Carlo simulation trials that each began by selecting 83,202 and 75,327 random SNPs displayed as many as 11,037 nominally significant results in both samples (p<0.00001). None of 10,000 permutation trials displayed results from permuted datasets that matched or exceeded the 11,037 SNPs actually observed (p<0.0001 by permutation analyses). These 11,037 SNPs thus provide the “reproducibly positive SNPs” for analytic approach (1).

**Figure 2:**
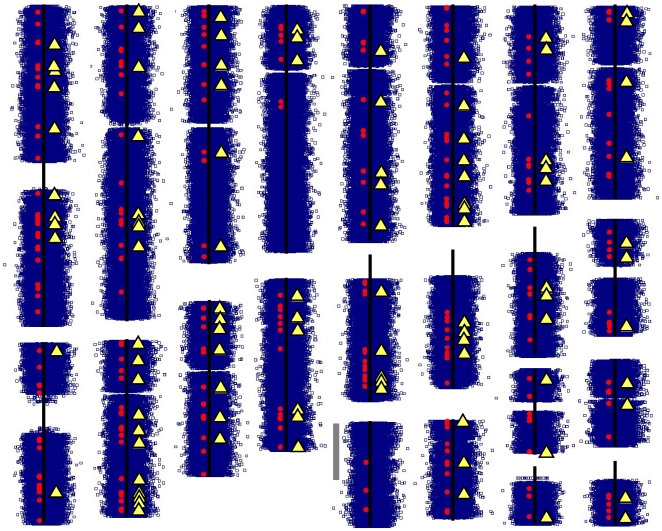
Chromosomal distributions of abuser/control t values, clustered positive SNPs, and candidate positive genes (Table 1). *Blue boxes:* t values of the abuser control differences from 870,000 SNPs studied here. Values from European-Americans: right side, from African-Americans: left side. *Red circles:* Positions of the SNPs whose data yield clustered positive values. *Yellow triangles:* positions of clustered positive results that support genes listed in Table 1. Scale bar (grey): 25 Mb.

The reproducibly-positive SNPs identified by abuser/control comparisons in both European- and African-American samples cluster together in small chromosomal regions (Fig. 2) to extents much greater than anticipated by chance. 937 of the reproducibly-positive SNPs from approach (1) lie in 271 clusters of ≥3 SNPs that are separated from each other by ≤25kb. This degree of clustering was never identified by chance (Monte Carlo p<0.00001). These clusters of reproducibly positive SNPs from analytic approach (1) identify 104 genes (Tables 1, 2). Randomly selected SNPs never cluster by chance within genes to the extent observed here (Monte Carlo p<0.00001).

**Table 1:** Results of “converge first then cluster” (approach 1) and “cluster first then converge (approach 2) analytic strategies applied to substance dependence vulnerability datasets described herein.

*#SNPs*		*Clustered SNPs*			*Number of genes*	*Overlap with 104 genes*
*clust*	*dist*	*AfAm*	*EuAm*	*converg*		
**CONVERGE FIRST, THEN CLUSTER (1)**						
**3**	**25,000**			**937**	**104**	**104**
3	10,000			299	37	37
4	25,000			328	31	31
4	10,000			86	10	10
**CLUSTER FIRST, THEN CONVERGE (2)**						
3	25,000	55,507	47,614	18,552	1,546	104
3	10,000	29,009	25,523	5,353	802	79
4	25,000	44,881	37,927	12,562	1,015	92
**4**	**10,000**	**17,849**	**15,779**	**2,142**	**341**	**52**

Columns list the numbers of SNPs that display abuser vs control differences with nominal p<0.05 (nominally positive) and lie in clusters, the maximal distance between nominally positive SNPs that is considered to indicate clustering, the numbers of clustered, nominally positive SNPs in African American samples, the numbers of clustered, nominally positive SNPs in European American samples, the numbers of “convergent” SNPs that display nominally positive results in both samples, the fraction of “convergent” SNPs that are likely to be true positives, on average, based on comparison with randomly chosen SNPs that are selected for similar convergence analyses (data not shown), the numbers of genes identified by the clusters of nominally positive SNPs and the overlap between the genes in each set and the 104 genes identified by the preplanned criteria used for primary analysis, using approach 1 with 3 SNP and 25kb intervals (*boldfaced*). The primary comparison set from approach (2) is also listed in boldface, based on the similar fraction of true positives anticipated using these criteria. We summarize these data in Fig. 2.

**Table 2:** Genes and classes of genes that contain clustered positive SNPs using the principal, preplanned analyses with criteria noted in Table 1.

				1: converge then cluster		2: cluster then converge		dbGAP support
*class/gene*	*chr*	*kbp*	*gene description*	*#SNPs*	*p*	*#SNPs AA/EA*	*p*	*# SNPs AA/EA*
*cell adhesion related*								
CDCP1	3	45098	CUB dom cont prot 1	3	0.021			
FHIT*	3	59710	fragile histid triad	5	0.022	29/62	0.003	38/24
ODZ2	5	166644	odd Oz/ten-m hom 2	3	0.091	21/23	0.011	4/0
CSMD1*	8	2782	CUB Sushi mult dom 1	10	0.004	117/137	0.001	84/55
CSMD3	8	113304	CUB Sushi mult dom 3	4	0.033			4/5
CD274	9	5440	CD274 molecule	5	0.002	7/6	0.013	
PCDH15	10	55250	protocadherin 15	3	0.094			
CTNNA3*	10	67349	α 3 catenin	3	0.158	29/6	0.059	0/20
NRXN3	14	77939	neurexin 3	3	0.134	18/5	0.110	4/0
SEMA6D	15	45797	semaphorin 6D	7	0.001	14/7	0.005	
THSD4	15	69220	thrombospondin I dom 4	3	0.067			
CDH13*	16	81218	cadherin 13	3	0.104	76/65	0.001	0/5
DSCAM*	21	40306	Down synd cell adh mol	4	0.026	23/36	0.003	8/0
*DNA/RNA handling*								
CHD1L	1	145180	chrdom h'case DNA bind1L	3	0.018			5/0
DDX1	2	15649	DEAD box polypept 1	3	0.019			
PRPF4	9	115077	pre-mRNA proc fact 4 hom	3	0.012			
PIWIL1	12	129388	piwi-like 1	3	0.019			
POLR1D	13	27094	RNA pol I polypep D	3	0.019			
SAMD4A	14	54104	ster a motif dom 4A	3	0.035			
RAD51L1	14	67356	RAD51-like 1	3	0.073	4/10	0.112	0/8
*enzyme*								
MKNK1	1	46795	MAP kin interact S/T kin 1	3	0.019			
AGBL4	1	48822	ATP/GTP binding protL 4	6	0.008	10/8	0.040	7/0
NME7	1	167368	nucleoside-diP kin	14	0.001	22/25	0.001	0/8
QSOX1	1	178390	quiescin Q6 SH ox'ase 1	3	0.019			7/0
PRKCE	2	45732	protein kinase C epsilon	3	0.059	13/22	0.002	12/0
LASS6	2	169021	ceramide synthase 6	3	0.042			
TMPRSS7	3	113236	serine TM protease 7	3	0.018			
EHHADH	3	186391	3-OHAc coA dehydrog'ase	3	0.022			4/4
GBA3	4	22303	acidic ß glucosidase 3	6	0.003	5/5	0.042	
PDE1C*	7	31795	calmod-dep P-diest'ase 1C	3	0.039			
MSRA	8	9949	methionine SO red'ase A	3	0.047			
ADARB2	10	1218	RNA-spec A deam'ase B2	3	0.061			5/9
SLK	10	105717	STE20-like kinase	3	0.019			
PRKCH	14	60858	protein kinase C eta	5	0.008			
XYLT1	16	17108	xylosyltransferase I	3	0.048	4/11	0.043	0/4
*ligand*								
CXCL14	5	134934	chemokine ligand 14	4	0.005	5/5	0.020	
*protein handling/modification*								
TSSC1	2	3171	tumor sup subtrans cand 1	4	0.009	6/6	0.034	
FKBP15	9	114967	FK506 binding protein 15	4	0.006			
HSPA12A	10	118419	HSP 12A	3	0.020	10/15	0.004	
BRWD2	10	122600	bromodom WD dom 2	3	0.022			
DOCK1	10	128658	ded cytokinesis 1	3	0.051			0/7
PACS1	11	65594	Pfurin sort prot 1	4	0.010	7/9	0.019	
CCDC91	12	28301	coiled-coil dom 91	10	0.001	5/17	0.012	
XPO6	16	28016	exportin 6	4	0.006	12/5	0.011	
PMAIP1	18	55718	PMA-induced prot 1	3	0.011	4/4	0.041	
*receptor*								
OPRD1	1	29011	δ opioid rec 1	6	0.001	10/6	0.011	
PLA2R1	2	160506	Pipase A2 rec 1	4	0.006			
GRM7*	3	6877	metabo glut rec 7	3	0.083	4/5	0.226	9/34
GRIK2	6	101953	ino glut rec kainate 2	3	0.071	5/4	0.191	10/0
OR51E1	11	4630	olfactory rec 51 E 1	3	0.011			
LDLRAD3	11	35922	low dens lipoprot rec A 3	3	0.042	13/6	0.020	6/0
GRM5	11	87880	metabo glut rec 5	5	0.011	20/7	0.015	5/0
GRIA4	11	104986	ino glut rec AMPA 4	3	0.048			
COLEC12	18	309	collectin sub-fam 12	3	0.031			
INSR	19	7067	insulin rec	3	0.032			
*signaling*								
BCAR3	1	93799	br ca anti-est res 3	3	0.025			
TTC21B	2	165905	tetratricopept rep dom 21B	4	0.020	8/4	0.118	10/12
TIAM2	6	155453	T-cell lymph inv met 2	3	0.032			
FAM126A	7	22949	fam seq similar 126 A	3	0.022	6/4	0.032	
ANO4	12	99712	anoctamin 4	3	0.041			0/5
APPL2	12	104091	pY inter PH dom leu zip 2	3	0.021			9/0
*structure*								
INADL	1	61980	InaD-like	3	0.048			0/9
LIMCH1	4	41057	LIM calpon homol dom 1	3	0.045			
DNAH8	6	38798	dynein h polypept 8	4	0.012	5/4	0.090	4/0
MYO6	6	76515	myosin VI	3	0.029	4/4	0.091	
AKAP7	6	131508	A kinase anchor prot 7	4	0.008	4/7	0.040	
SYNE1	6	152484	spectrin rep nuc env 1	3	0.058	26/5	0.007	6/7
CADPS2	7	121746	Ca-dep act prot secret 2	3	0.063			
CHCHD3	7	132120	coil-coil-helix dom 3	3	0.039	7/4	0.064	
MPP7	10	28382	palmitoyl memb prot 7	3	0.033			
ABLIM1	10	116180	actin bind LIM protein 1	3	0.040			
PARVA	11	12355	a parvin	4	0.010	4/12	0.018	
CSRP3	11	19160	C G-rich prot 3	3	0.016			
FARP1	13	97593	FERM RhoGEF pleckst 1	3	0.043			
MYO5C	15	50271	myosin VC	3	0.023	4/9	0.027	
FHOD3	18	32131	formin homol 2 cont 3	3	0.056			0/12
*transcription regulation*								
PBX1	1	162795	pre-B-cell leukemia TF 1	5	0.005			
AFF3	2	99530	AF4/FMR2 3	3	0.063			
CSRNP3	2	166137	cys-ser-rich nuclear prot 3	3	0.029	3/3	0.081	6/0
ZNF804A	2	185171	zinc finger protein 804A	4	0.011	4/4	0.141	
ZNF385D	3	21437	zinc finger protein 385D	3	0.042	12/4	0.033	0/5
ZNF366	5	71774	zinc finger protein 366	8	0.001	7/11	0.008	
ETV6	12	11694	ets variant gene 6	3	0.038	4/25	0.004	
KLF12	13	73158	Kruppel-like factor 12	4	0.015	4/7	0.094	
ZNF606	19	63180	zinc finger protein 606	3	0.017			
LDOC1L	22	43267	L zip down-reg ca 1-L	3	0.012	5/4	0.028	
*transport*								
ATP1B1	1	167342	Na/K transpor ATPase ß 1	4	0.004	4/4	0.053	0/3
SLC45A2	5	33980	solute carrier 45 2	4	0.005			11/0
CFTR	7	116907	ATP-binding cassette C 7	3	0.033			5/0
XKR4	8	56177	Kell blood gp comp 4	3	0.051	8/8	0.043	
SLC2A13	12	38435	solute ligand carrier 2 13	6	0.004	17/4	0.019	0/8
ABCC4*	13	94470	ATP-binding cassette C 4	4	0.012	13/4	0.023	23/30
SLC10A2	13	102494	solute ligand carrier10 2	3	0.014	12/7	0.005	0/8
*unknown*								
KIAA1276	4	17242	KIAA1276 protein	3	0.029			
FLJ44606	5	126411	FLJ44606	3	0.016	5/4	0.034	
FAM184A	6	119322	fam seq sim 184 A	3	0.028			
BRP44L	6	166698	brain protein 44-L	3	0.015			
FRMD4A	10	13725	FERM dom 4A	3	0.071	4/23	0.021	5/13
C10orf11	10	77212	Ch 10 ORF 11	3	0.077			
C10orf82	10	118413	Ch 10 ORF 82	3	0.013	5/6	0.017	
C19orf18	19	63161	Ch 19 ORF 18	3	0.013			
MACROD2	20	13924	MACRO dom 2	3	0.175	57/22	0.004	6/8
C20orf70	20	31219	Ch 20 ORF 70	3	0.014			
RHBDD3	22	27985	rhomboid dom 3	3	0.014			

These “converge then cluster” genes thus each contain three or more SNPs that display nominally significant allele frequency differences between both European-American (EA) and African-American (AA) polysubstance abuser *vs* control comparisons that cluster within <25kb of each other and lie within the gene's exons or within +/−10 kb 3′ or 5′ flanking sequences. Genes are grouped by the class of the function to which they contribute. The numbers of reproducibly positive SNPs that lay in clusters within the gene's exons and in 10 kb genomic flanking regions are noted. Chromosome number and initial chromosomal position for the cluster (bp, NCBI Mapviewer Build 36.1) are listed. “Approach 2/Cluster then converge” genes that were identified by clusters of at least 4 nominally positive SNPs that lay within 10kb of each other and lay within the gene for each sample are listed in the column labeled “**2: cluster then converge**”. Asterisk identifies genes also identified in [16]. P values are based on 10,000 Monte Carlo simulation trials in which the number of times randomly-selected segments of the genome that lie within genes are assessed for the same features displayed by the actual gene identified. Relevant rs numbers for SNPs are listed in Table S2. dbGAP support lists the numbers of SNPs in the same genes that display nominally-significant differences between cocaine-dependent and nondependent control AA and EA samples from 1M SNP Illumina individual genotyping of samples from COGA, FSCD and COGEND samples as described in dbGAP (http://www.ncbi.nlm.nih.gov/sites/entrez?Db=gap).

We would anticipate the observed, highly significant clustering of SNPs that display nominally positive results if many of these reproducibly positive SNPs lay near and were in linkage disequilibrium with functional allelic variants that distinguished substance dependent subjects from control subjects, but not if they represented chance observations. The Monte Carlo p values noted here are likely to receive contributions from both the extent of linkage disequilibrium among the clustered, nominally positive SNPs and the extent of linkage disequilibrium between these SNPs and the functional haplotype(s) that lead to the association with substance dependence.

### “Cluster Then Converge”

Non-template analytic approach (2) “cluster then converge” also assessed the significance of the 83,202 and 75,327 SNPs that displayed nominally positive results in African-American and European-American samples, respectively. 17,849 SNPs from African American and 15,779 SNPs from European American samples provide clusters, within each sample, of at least 4 nominally positive SNPs that lie within ≤10kb of each other. 2,142 of these SNPs lie within clusters that identify the same genes in both African- and European-American samples (Tables 1, 2). The 341 genes that are identified in this way (Table S1) are never identified by chance, using this approach, in 100,000 Monte Carlo II simulation trials (p<0.00001)([36] and Materials and Methods). The genes identified using approach (2) overlap with ½ of the genes identified using approach (1) (Table 2).

### Controls for Alternative Hypotheses

Controls for occult stratification do not appear to provide convincing alternative explanations for the data obtained here. Only 22 of the 937 clustered, reproducibly positive SNPs that we identify here using approach (1) also display sizable allele frequency differences based on ethnicity. Since we would have expected 24 by chance, it appears highly unlikely that stratification based on racial/ethnic differences between each abuser and corresponding control samples provides a major basis for the addiction-associated allelic variants identified herein. Principal components analyses identify robust principal components that cleanly separate African-American from European-American samples and account for about 94% of the variance when both samples are analyzed together (*data not shown*). However, there is significant distinction between substance dependent *vs* control pools based on additional principal components that are orthogonal to those that distinguish individuals with African *vs* European heritages. Analyses of the likelihood that substance dependent samples would be distinguished from control samples by this principal component based on chance yield p = 0.00003 and p = 0.057 probabilities in European- and African-American samples, respectively (*data not shown*).

Assay noise also fails to provide a convincing alternative explanation for the data reported herein. When we examined the overlap between the clustered positive SNPs and the 10% of the SNPs for which the correlations between observed and expected values in validating studies were poorest, we found about as many (15) as we would have expected to find by chance (13). The t tests used for assignment of primary nominal significance also correct for assay variability. There is thus no indication that assay noise provides the sole basis for the addiction-associated allelic variants identified herein.

### Overlap between Genes Identified Here and Those Identified by Other Previously Reported GWA Datasets

The genes identified using approaches (1) or (2) each overlap with genes identified in several other GWA datasets for substance dependence, based on Monte Carlo simulations ([36] and Materials and Methods). There are significant overlaps with data from: a) 600k GWA studies of a subset of these polysubstance abusers (p<0.001) [16], b) 500–600k GWA data from studies of methamphetamine dependent Japanese samples [17] (p = 0.04), c) 100k GWA data from studies of alcohol dependence in European-Americans [15] (p = 0.0003) and d) 38k data from comparisons between more frequently nicotine dependent *vs* less frequently nicotine dependent groups of smokers [37], [38] (p = 0.02). We also identify substantial overlap with individually genotyped data from dbGAP samples of cocaine dependent *vs* control individuals (Table 2). This overlap is even more impressive when we compare the genomic regions identified by clustered, nominally significant results from samples of the same racial/ethnic group (Drgon et al, in preparation).

### Preferential Brain Expression of Genes Identified Here

We evaluated evidence for preferential brain and brain regional expression patterns of the 104 genes identified in Table 2. Brain libraries contained at least two expressed sequence tags (ESTs) that corresponded to 79% (82/104) of the genes in Table 2. These ESTs came from amygdala (255), adult brain (736), developing brain (1243), caudate (striatum) (63), cerebellum (375), cerebral cortex (52), hippocampus (554), hypothalamus (274), medulla oblongata (31), substantia nigra (43), subthalamic nucleus (38), thalamus (187), corpus callosum or other white matter (72) and peripheral nerve (97). Levels of expression for this set of genes (compared to all genes) displayed nominal significance in thalamus, hippocampus, amygdala, cerebellum, substantia nigra, hypothalamus and whole brain (p = 0.002, 0.003, 0.004, 0.005, 0.02, 0.03 and 0.02, respectively). After Bonferroni corrections, values for thalamus and hippocampus (p = 0.032 and 0.045, respectively) retained significance. Assessments of the “more reliable” subset of ESTs revealed significant over expression in hippocampus and whole brain (corrected p *ca* 0.01 for each).

## Discussion

### “Replication” of Genome Wide Association Results

Genome-wide association data of increasing richness is available for a number of complex disorders. Several of these GWA datasets contain relatively robust results at “oligogenic” loci that can also be identified by linkage-based approaches [39]–[42]. Even moderately secure GWA identification of “polygenic” influences on disease, however, is likely to require replicated data from multiple independent samples.

There have been no unanimous criteria for declaring such replication in circumstances in which no SNP provides “genome wide significance” with the same phase of association in “template” GWA analyses of data from multiple independent samples. Replication of nominally significant associations for the same SNP (approach (1), here) is among the criteria most used to date [43], [44]. This “nontemplate” GWA analytic approach is likely to perform best when large association signals are found in each independent sample, when the same SNP sets are studied in each, when the disease exhibits little locus heterogeneity and when there are good matches between the fine patterns of linkage disequilibrium of the samples being studied and the reference samples (commonly, Hap Map) used to infer the underlying patterns of linkage disequilibrium. Few “replication” samples manifest all of these features. Although the current European- and African-American samples were recruited in parallel and evaluated with the same SNPs, the racial/ethnic differences between the participants suggest that the samples are likely to manifest differences in fine patterns of linkage disequilibrium and in phase of association at many loci. Apparent replication “failures” using approach (1) could thus relate to sample-to-sample differences in fine patterns of linkage disequilibrium and/or different amounts of information provided by markers with population-specific differences in allele frequencies. Allelic heterogeneities could also make contributions. Conceivably, genes for which the Monte Carlo p values determined by approach (2) are much stronger than the estimates based on approach (1) might provide interesting candidates for such allelic heterogeneity. Positive findings in the “cluster than converge” approach that are supported by evidence from other studies might be especially attractive candidates. NRXN3 and GABBR2 were identified using this approach and also in linkage studies of opiate dependence [45] and association studies of nicotine dependence [6], [46], for example.

### “Converge Then Cluster” and “Cluster Then Converge” Approaches

Results from approaches (1) and (2) share a number of potential strengths. These datasets provide significant concordance with each other, with previous GWA datasets for substance dependence, and with dbGAP data that has been available only after the results of the current study were analyzed. The arrays used here provide power to identify many of the genes, especially genes of smaller size, which could not have been identified in previous GWA studies that used analysis (1) and lower density arrays [25].

Monte Carlo methods allow us to test the probabilities of chance clustering of nominally positive SNPs and the chance of convergence between clusters identified in one sample with clusters identified in other samples. Our Monte Carlo approaches deploy an empirical method that uses the existing dataset as a source for randomly selected SNPs for each Monte Carlo trial. The results of these simulations, supported by data derived from permutation, principal components and other analyses, provide strong overall confidence that these results are not due to chance. By contrast, these approaches provide absolutely unequivocal identification for few individual SNPs. This lack of unequivocal identification of individual SNPs is consistent with the current polygenic working models for the genetic architecture of vulnerability to substance abuse [25], [47].

### Differences between “Non-Template” and “Template” GWA Approaches

Validation studies provide evidence for excellent correlations between individually genotyped and pooled allele frequency assessments. However, the current “nontemplate” approaches and data do provide a number of differences from the “template” genome wide association approaches used in recent reports from larger projects that employ individual genotyping in studies of legal phenotypes, for example the GWA studies of complex phenotypes currently listed in dbGAP (http://www.ncbi.nlm.nih.gov/sites/entrez?Db=gap). 1a) The samples for these studies are typically compiled from recruitments at many sites. The studies typically combine subjects recruited based on multiple sets of criteria for selection. There is no indication of the fraction of individuals approached who consented. Cases are compared to controls who were almost always recruited and collected at different times and are either uncharacterized or evaluated using methods different from those applied to cases. 1b) By comparison, all of the dependent and control individuals studied in the present sample were collected at the same site, recruited in ways that result in virtually all candidates consenting to participation, and assessed using the same instruments. Controls are thus characterized in such a manner that they each provide a contrast with the dependent cases. 2a) Genotyping data in dbGAP typically uses data from single microarrays that are hybridized with fluorescently-labeled DNAs prepared from DNAs from single individuals, hybridization intensities assessed, and genotype calls made based on Bayesian and other algorithms using data from the ratios of hybridization intensities to probes that are complementary to alternative allelic forms of each SNP. Quality control efforts for samples and SNPs use predetermined algorithms for hybridization signal differences and analyses of Hardy-Weinberg equilibria. Few studies provide test-retest data to evaluate the fraction of genotypes that are replicable, however. 2b) The current approach uses data from three microarrays that are hybridized with three distinct preparations of fluorescently labeled DNA that are carefully prepared from pools of DNA from 20 individuals. Quality control evaluations come chiefly from assessment of the array-to-array variation in hybridization intensities noted for replicate experiments, as well as assessments of pool-to-pool variation, as noted here. These assessments thus do not measure the features that are assessed by Affymetrix or Illumina software packages, but rely on estimates of variability in relation to the signals obtained. The 0.98+ correlation between observed and expected allele frequencies provides a modest difference from a set of perfectly accurate individual genotypes. 3a) The sample sizes in studies currently listed in dbGAP are larger, with an average size of 2,155 cases and controls 3b) The sample size here of 1,620 is divided into two separate case *vs* control comparisons, providing, as we note, moderate power to detect replicable modest-sized effects and lower power to detect very small effects. True effects that provide nominal statistical significance in clustered SNPs in only one sample represent false-negative findings in our first analysis. 4a) Many of the results of individually-genotyped studies represented in dbGAP are analyzed based on the assumption that the detailed haplotype structures identified in data from CEPH and Yoruban individuals will provide accurate representations of the haplotypes identified in European-American and African-American samples identified far from Utah. 4b) The approach that we use here is based on distances between SNP genomic markers, rather than assumptions about the extent to which the exact haplotype structure of these reference populations will be maintained in the samples that we have studied. 5a) Dense individual genotypes provide the opportunity for unequivocal matching with DNA databases; 5b) Pooled genotypes provide a much stronger barrier for matching with DNA databases, which is an enhanced consideration in studies of illegal behaviors. 6a) “Template” analyses focus on strength of association for individual SNPs; 6b) The current analyses assume that most *bona fide* associations that are based on phenotypic differences will be present at multiple nearby SNPs. In any single sample, many of the clusters of positive findings at nearby SNPs could be due to stochastic differences in haplotype frequencies between cases and controls that are not related to phenotype. As the same chromosomal regions are identified by more and more independent samples, however, the likelihood that such identification is due to stochastic differences in haplotype frequencies that are unrelated to phenotype declines sharply. Assessment in multiple, independent samples, as we perform here, provides the best control for stochastic differences in haplotype frequencies that might be expected, by chance, between any single case and control samples. 7a) “Template” GWA analyses that focus on single SNPs may provide modest biases toward identification of large genes that contain many SNPs; 7b) The current analyses require nominally significant associations for multiple SNPs that lie within narrow chromosomal regions. A number of the smallest genes cannot be identified by this approach [25], in ways that might lead to an even more prominent bias toward identification of larger genes in this way; 8a) “Template” GWA approaches focus on metaanalyses as means to evaluate convergence of data from single SNPs across many independent samples; 8b) As more such data becomes available, metaanalyses can be applied to the current results. However, in the relative absence of other GWA datasets (*but see below*), metaanalyses are of more limited utility.

### Genes Also Identified in Other Studies of Addiction

Despite the differences in approaches, primary substance of abuse and/or genetic background, however, there is significant evidence that, compared to chance, the current results identify more of the same genes and chromosomal regions that are also identified by a number of independent datasets that compare substance dependence phenotypes to controls. These include the fits between the current nontemplate analyses of data from pooled African- and European-American samples and previously-reported pooled results from Asian methamphetamine dependence samples as well as individual genotyping results that compare dependent *vs* nondependent smokers. Analyses of cocaine dependence *vs* control data that has become available on dbGAP after our analyses were completed provide support for 162 of the clusters of nominally-positive result from the European-American samples reported herein by clusters of at least 4 SNPs that display p<0.05 nominal significance in this dbGAP data (Monte Carlo p<0.0001) (Drgon et al, in preparation). Data for African-American samples provides support for 147 of the clustered, nominally significant observations from our current report (Monte Carlo p<0.0001) (Drgon et al, in preparation).

Our identification of some SNP markers whose allelic frequencies distinguish controls from addicts of different ethnicities supports “common disease/common allele” genetic architecture for significant portions of addiction vulnerability [48] based on relatively old allelic variants. The good fits between results from ethnically-matched samples from the current data and dbGAP samples also support the idea that many addiction vulnerability variants are likely to display substantial differences from one human population to another (Drgon et al, in preparation). In our current data, the same phase is not detected for many of the SNP associations noted in samples of different racial/ethnic backgrounds. Although we believe that this is a likely consequence of the differences in detailed linkage disequilibrium between the SNP markers and the actual pathogenic allelic variants at many of these chromosomal loci, some of these phase differences are also likely to reflect results that co-occur in the two independent samples by chance.

The convergent data derived from studies of individuals with addictions to substances in several different pharmacological classes support the idea that many of these allelic variants enhance vulnerability to many addictions. These results do not exclude additional contributions to addiction vulnerability from genomic variants that influence vulnerability to specific substances or variants that are found only in specific populations, however.

### Classes of Genes Identified Here

We focus on identification of genes. Although associations in chromosomal regions that do not contain annotated genes also provide interesting results, the genes that we identify in the present work provide a number of interesting views of addiction. 1) More are represented among cDNAs cloned from brain libraries than is the case for all human genes. While all do not display at least two cDNAs in at least one brain library represented in dbEST, it seems likely that many of the remaining genes are expressed at low levels and/or in small brain regions that are not adequately represented in many of these libraries. 2) The results from dbEST studies of the expression of these genes focus attention on expression in hippocampus, which manifests interesting roles in mnemonic processes in ways that may provide clues to the pathophysiology of human addiction. 3) These genes do not overlap, to extents greater than expected by chance, with genes listed in the Knowledgebase for Addiction Related Genes (KARG), a recent compilation of literature and database information concerning addiction related genes [49]. 4) Gene ontology searches (BioBase) reveal “Biological Process” terms that displayed the strongest trends toward overrepresentation (compared to Bonferroni-corrected p = 7.8×10^−5^) among human gene classes: glutamate signaling pathway (p = 0.00019); auditory receptor cell differentiation (p = 0.0047); endocytosis (p = 0.0065); sex determination (p = 0.0081); inner ear receptor cell differentiation (p = 0.0081); endothelial cell proliferation (p = 0.0088); synaptic transmission (p = 0.01); mechanoreceptor differentiation (p = 0.01); adult locomotor behavior (p = 0.01) and regulation of synaptic plasticity (p = 0.01). Mouse data for glutamate signaling pathway (p = 8.2×10^−6^) provides the strongest p values that do exceed the Bonferroni correction (data not shown).

Identification of cell adhesion molecule genes, which are represented in several of the above mentioned gene classes (Table 2) continues to focus our attention on roles in addiction for mechanisms for establishing and regulating neuronal connections [50]–[52]. These data accord well with prior results that link substance dependence to 5′ NrCAM variants that alter levels of expression and to 3′ NRXN3 variants that alter relative levels of splicing isoforms [45], [53]. It is important to note that these cell adhesion genes are generally large, providing more opportunities for allelic variants that could alter their functions in a number of fashions. It is interesting to note that recent analyses of all reported genome wide association datasets also identified overrepresentation of cell adhesion molecule genes [54].

We also identify genes that are likely to be readily targeted and modulated by drugs, and thus provide potential pharmacotherapeutic targets for addictions. G protein coupled receptors that include the δ opioid receptor and metabotropic glutamate receptor 5 display rich pharmacologies and substantial ties to addiction through abundant evidence from pharmacologic, knockout mouse and other approaches. Glutamatergic systems are also implicated by identification of “druggable” metabotropic 7, GRIK2 kainate and GRIA4 AMPA glutamate receptors, as well as several cell adhesion molecules that are associated with classical synapses. Known small molecules and/or drugs act at many of the other receptors, enzymes and transporters listed in Table 2.

### Genes Also Identified in Studies of Heritable, Co-Occurring Phenotypes

A number of the genes identified in this work are also identified in genome wide association and/or candidate gene datasets for heritable disorders or phenotypes that co-occur with addictions [25]. Differences in memory and cognitive systems have long been identified in addicted individuals; we have identified significant overlaps between the addiction associated genes listed below and the results of GWA studies for individual differences in cognitive abilities. NRXN3 and a number of other genes listed in Table 2 also display associations with the memory-associated neurodegenerative disorder, Alzheimer's disease (Hishimoto et al, submitted). Frontal lobe volumes are smaller in several studies of substance dependent individuals or their offspring; there is significant overlap between GWA results for frontal lobe volumes and addiction [25]. Overall GWA results for bipolar disorder, in which a majority of individuals may abuse or be dependent on addictive substances, overlap with addiction GWA data [25]. There is also a significant overall overlap between addiction vulnerability GWA data and the genes identified in studies of success in smoking cessation [25].

### Conclusions

The findings presented here promise to add to the ongoing consideration of methods for comparing GWA datasets as they enhance understanding of genetic underpinnings of human addiction. For addictions, as for many complex disorders, such data provides an increasingly rich basis for improved understanding and for personalized prevention and treatment strategies.

## Materials and Methods

### NIDA Research Volunteers

Research volunteers who came to the NIDA research facility in Baltimore, Maryland between 1990 and 2007 in response to advertisements and referrals from other research volunteers provided written informed consents, self-reported ethnicity data, drug use histories *via* the Drug Use Survey and DSMIII-R or IV diagnoses and were reimbursed for their time at different rates during this period (currently about $120 total) as previously described [13], [55], [56]. DNA in 81 pools sampled: a) 400 unrelated European-American “abusers” (mean age and standard deviation = 34 (+/−2), 0.79 male) with heavy lifetime use of illegal substances and, for virtually all, DSMIII-R/IV dependence on at least one illegal abused substance b) 280 “control” European-Americans (mean age 32 (+/−3), 0.6 male) who reported no significant lifetime use of any addictive substance, c) 700 African-American abusers (mean age 34 (+/−3), 0.75 male) and d) 240 African-American controls (mean age 35 (+/−5), 0.43 male) [13], [14], [16]. The modest differences between mean ages of dependent and control individuals would be expected to yield virtually no differences in cumulative lifetime probabilities of developing dependence on one or more illegal substances, based on data from the National Survey of Drug Use in Households (http://oas.samhsa.gov/nsduh/2k7nsduh/2k7Results.cfm#TOC).

DNAs were assessed in pools since this: 1) provided us with the maximal ability to protect the genetic confidentiality of subjects who volunteered for study of genetics of illegal behaviors, 2) allowed us to utilize DNAs from individuals who consented to participation in this study during time periods when consents did not explicitly describe studies using high densities of DNA markers, 3) allowed us to utilize DNAs from individuals whose consents explicitly committed us to use DNA pooling methods wherever possible to maximize protection of their genetic confidentiality, 4) allowed us to use methods that we have developed and validated in this and in previous work and 5) reduced costs. Most of these subjects would thus not have been available for studies that assessed substantial numbers of polymorphisms using individual genotyping.

DNA was prepared from blood [13], [55], [56]. Genotyping and primary assessments of genotyping were performed by investigators blinded to clinical diagnoses. DNAs from groups of 20 individuals of the same ethnicity and phenotype were carefully quantitated and combined. This number of individuals/pool was selected since we have extensively validated use of pools of this size [13]–[17], [37], [38] with respect to statistical power as well as the advantages and disadvantages of pooling noted above. Hybridization probes were prepared as described (Affymetrix assay 6.0, [15]). For each pool 150 ng of pooled DNA was processed, labeled and hybridized to Affymetrix 6.0 arrays according to the instructions of the manufacturer (Affymetrix, Santa Clara CA) and [13]–[15]. Quality controls for assays were performed as recommended (Affymetrix, Santa Clara CA) and (Fig. S2). Features of this portion of methods are depicted in Fig. S1.

### Identification of Nominally-Positive SNPs

For NIDA individuals allele frequencies for each SNP in each DNA pool were assessed based on hybridization to the 3–4 “perfect match” cells on each of three arrays, as described [14], [15]. We validated this approach (Fig. 1) [14], [15]. The intensities of the highest and lowest 5% of features on each array were monitored and the variances in signal between replicate hybridizations of DNA from each pool and between hybridization signals from pools of the same phenotype and ethnicity were assessed. For the detection of nominally positive SNPs we averaged the “perfect match” data for each SNP on each array, derived the arctangent of the ratio between hybridization intensities for A and B alleles, averaged the arctan A/B values for the three replicate arrays, divided the mean arctan A/B ratios for abusers by the mean arctan A/B ratios for controls to form an abuser/control ratio for each SNP, and generated a “t” statistic for the differences between arctan A/B in abusers and controls with corresponding p values (see Fig. S1 for this portion of the “cluster then converge” analysis and Fig S2 for initial quality control).

We deleted data from SNPs on sex chromosomes. This allowed us to combine data from male and female subjects and increase overall power. We also deleted data for SNPs whose chromosomal positions could not be adequately determined.

### Identification of Genes That Contained Convergent Data from Two Samples: 1) “Converge Then Cluster”

For these analyses, we identified “reproducibly-positive” SNPs that cluster in small genomic regions within genes (Table 1). These SNPs a) display t values with p<0.05 significance in both African- and European-American abuser *vs* control comparisons (we define this evidence for significant association in each of two independent samples as “reproducible”), b) cluster, so that at least three reproducibly-positive SNPs lie within 25kb of other reproducibly-positive SNPs, c) identify genes (Table 2, *legend*). The 104 genes in Table 2 are supported by reproducible clustered positive association data from the same SNPs in each of two samples. We identify subsets of these genes when we impose more stringent criteria for: a) ≤10,000 basepair distances between reproducibly-positive SNPs, and b) at least 4 nominally-positive SNPs per cluster (Table 1).

### Identification of Genes That Contained Convergent Data from Two Samples: 2) “Cluster Than Converge”

To provide complementary analyses, we began by identifying clusters of positive SNPs that fall within genes in each sample. We identify the genes that are tagged by at least one cluster of nominally positive SNPs from each of the two samples (Table 1). We focus on a set of criteria for approach (2) that produce about the same ratio of “true” genes to total genes (“true”+“chance”) (0.43 *vs* 0.47) as those likely to be observed using the criteria on which we focused in approach (1). The SNPs that we identify in this secondary analysis thus a) display t values with p<0.05 significance in one abuser *vs* control comparison, b) cluster, so that at least four of these positive SNPs lie within 10kb of each other in this abuser *vs* control comparison, c) identify the same genes as clustered positive SNPs from the other sample (Table 2, *legend*, and Table S1). Three hundred forty one genes are identified in this way (Table S1). Fifty-two of the 104 genes listed in Table 2 based on approach (1) are also identified after application of the criteria noted above and approach (2). See Fig. S1 (points 4–6) for details of the last steps of “cluster then converge” analyses:

The sets of results from approaches (1) and (2) were compared to those expected by chance using 100,000 Monte Carlo II simulation trials (below) ([36] and below, and Johnson et al, in preparation). For each of 100,000 simulation trials, a random set of SNPs was chosen by sampling randomly from a list that contained all SNPs studied. The randomly chosen SNPs were considered “pseudopositive” SNPs for that trial. The number of trials for which the results from “pseudopositive” SNPs subjected to our analytical procedure matched or exceeded the results actually observed from the SNPs identified in the current study was tabulated. Empirical p values were calculated by dividing the number of trials for which the observed results were matched or exceeded by the total number of Monte Carlo simulation trials. Similar Monte Carlo III approaches ([36] and below) sampled from a dataset of all gene sequences. These approaches allowed us to generate nominal p values for the observations made for each gene listed in Table 2.

Monte Carlo I assessments [36] thus provided p values for the extent to which clustering of nominally positive SNPs (from each of 2 samples in “converge then cluster” approach and from each sample in “cluster then converge” approach) differed from chance. Monte Carlo II assessments [36] provided p values for the degree to which clusters identified in one of the samples from the “cluster then converge” approaches were found in the same genes as those identified by clusters from the other sample. Monte Carlo III assessments [36] provided p values for the likelihood of finding of sets of nominally-positive SNPs in segments of each of the genes identified here (Johnson et al, in preparation).

Secondary analysis used permutation approaches. We randomized assignment of the phenotypes to data derived from the current SNPs and analyzed the data in each of the 10,000 permutation trials.

To assess the power of our current approach we used current sample sizes and standard deviations, power calculator PS v2.1.31 [57], [58] and α = 0.05. Results from “Gene detective” [25] provided a secondary power assessment.

To provide controls for the possibility that observed abuser-control differences were due to a) occult ethnic/racial allele frequency differences or b) noisy assays, we assessed the overlap between our results and SNPs that displayed the largest a) allele frequency differences between African-American *vs* European-American control individuals and b) the largest assay “noise”.

To seek patterns of human brain expression for the genes identified herein, we identified 846 human cDNA libraries constructed from brains with modest or no pathology in dbEST. We identified 1) all entries and 2) “more reliable” entries with correct genomic orientation and either evidence for polyA tail or spliced structure (CYL and GRU, in preparation). For each brain region, we assessed the *p*-value for over-representation of expression of the addiction-associated genes using hypergeometric distribution tests and false discovery rate (FDR) corrections, considering *Q*-values<0.05 as statistically significant.

### dbGAP Samples from the Family Study of Cocaine Dependence, COGA and COGEND Studies

Unrelated subjects who met DSM criteria for cocaine dependence and control subjects with no evidence for dependence on any addictive substance were assembled from three studies. Family study of cocaine dependence subjects were recruited from treatment centers close to St. Louis. Mo; 55% of contacted subjects participated. Community-based comparison subjects were recruited through driver's license records from the Missouri Family Registry and were matched to cocaine dependent subjects based on date of birth, ethnicity, gender, and zip code. Eighty percent of screened and eligible comparison subjects participated. Other participants came from individuals who participated in the collaborative study on the genetics of alcoholism and the collaborative study on the genetics of nicotine dependence. Dependent individuals displayed DSM dependence on cocaine as reflected in the dbGAP variable phv00066444.v1.p1. Controls displayed no DSM dependence on cocaine, nicotine, alcohol, marijuana, opioids or other drugs. We identified 481 dependent and 1053 control unrelated European-American and 516 dependent and 409 control unrelated African-American subjects for this analysis. Genotyping for these samples was performed using Illumina 1M SNP arrays at the Center for Inherited Disease Research (CIDR), with quality controls and principal components analysis (PCA) controls for racial/ethnic background available at the CIDR website (www.cidr.jhmi.edu). Genotypes from dependent and control individuals were selected from dbGAP files. p values for each SNP were based on χ^2^ tests. The resulting p values were then analyzed and compared to the results from the NIDA individuals by the “cluster then converge” method described above.

Gene Ontology analysis was performed in BioBase Systems Biology Environment (http://www.biobase.de) installed on http://helixweb.nih.gov/biobase.

## Supporting Information

Figure S1 Simplified schematics for methods used in the current analyses. A: Methodological schematic. First two lines denote separation of African-American and European-American samples into “case”, “control” and “other” phenotypes. Third line denotes pooling DNAs from groups of 20 individuals of the same racial/ethnic and phenotype group. Lines 4–6 denote analyzing DNA from each of the pools using three independent Affymetrix 6.0 array assays. B: Analytic schematic. Points 1–3 and “Manhattan plots” indicate analyses that identify SNPs with nominally-significant case vs control differences. Point 4a: Converge then cluster analysis. Point 4b: Cluster then converge analysis. Point 5 emphasizes gene-centered analysis used herein.
(0.07 MB PDF)

Figure S2 QQ plots for distribution of t values from African-American data (left) and European American data (right). Observed data from these experiments provides deviations from expected data generated from 10,000 t tests which were each run from a set of random values of the same size as those obtained from the true datasets. Deviations noted at the right side of these plots are likely to represent both a) true case vs control differences and b) nonnormal differences in the distribution of t values from the bona fide data. In the current “nontemplate” analyses, we use t values to identify the 5% of SNPs with the highest t values and subsequent testing with empirical statistics to assign overall levels of significance. Thus, any nonnormal component of this distribution is of less concern than it would be for “template” GWA analyses in which t values might be used as the primary determinant of (e.g., genome wide) significance.
(0.03 MB PDF)

Table S1 Genes identified by “cluster then converge” secondary analyses (2), as described in the text. These genes are thus each identified by clusters of four or more SNPs that display nominally significant allele frequency differences between polysubstance abuser vs control comparisons, cluster within <10kb of each other and lie within the gene's exons or within +/−10 kb 3′ or 5′ flanking sequences. Note that for this analysis, the same SNPs are not required to display nominal significance in each of the two samples. p values are based on 10,000 Monte Carlo simulation trials in which the number of times randomly-selected segments of the genome that lie within genes are assessed for the same features displayed by the actual gene identified. Some, but not all, of these genes are also identified by (1) converge then cluster analyses as noted in Table 1 and by clustered nominally-significant SNPs from dbGAP datasets, as noted in Table 2.
(0.04 MB PDF)

Table S2 List of RS numbers and chromosomal location identified as “positive” that are the basis for identification of the genes in Table 2 in the manuscript.
(0.03 MB PDF)
