# Supplementary material for: Genome Wide Association for Addiction: Replicated Results and Comparisons of Two Analytic Approaches
Source: PLoS One. 2010 Jan 21;5(1):e8832. doi: 10.1371/journal.pone.0008832 (PMC2809089; doi:10.1371/journal.pone.0008832)
Supplement: Figure S1 — Simplified schematics for methods used in the current analyses. A: Methodological schematic. First two lines denote separation of African-American and European-American samples into “case”, “control” and “other” phenotypes. Third line denotes pooling DNAs from groups of 20 individuals of the same racial/ethnic and phenotype group. Lines 4–6 denote analyzing DNA from each of the pools using three independent Affymetrix 6.0 array assays. B: Analytic schematic. Points 1–3 and “Manhattan plots” indicate analyses that identify SNPs with nominally-significant case vs control differences. Point 4a: Converge then cluster analysis. Point 4b: Cluster then converge analysis. Point 5 emphasizes gene-centered analysis used herein. (0.07 MB PDF) [file pone.0008832.s001.pdf]

Figure S1.

A

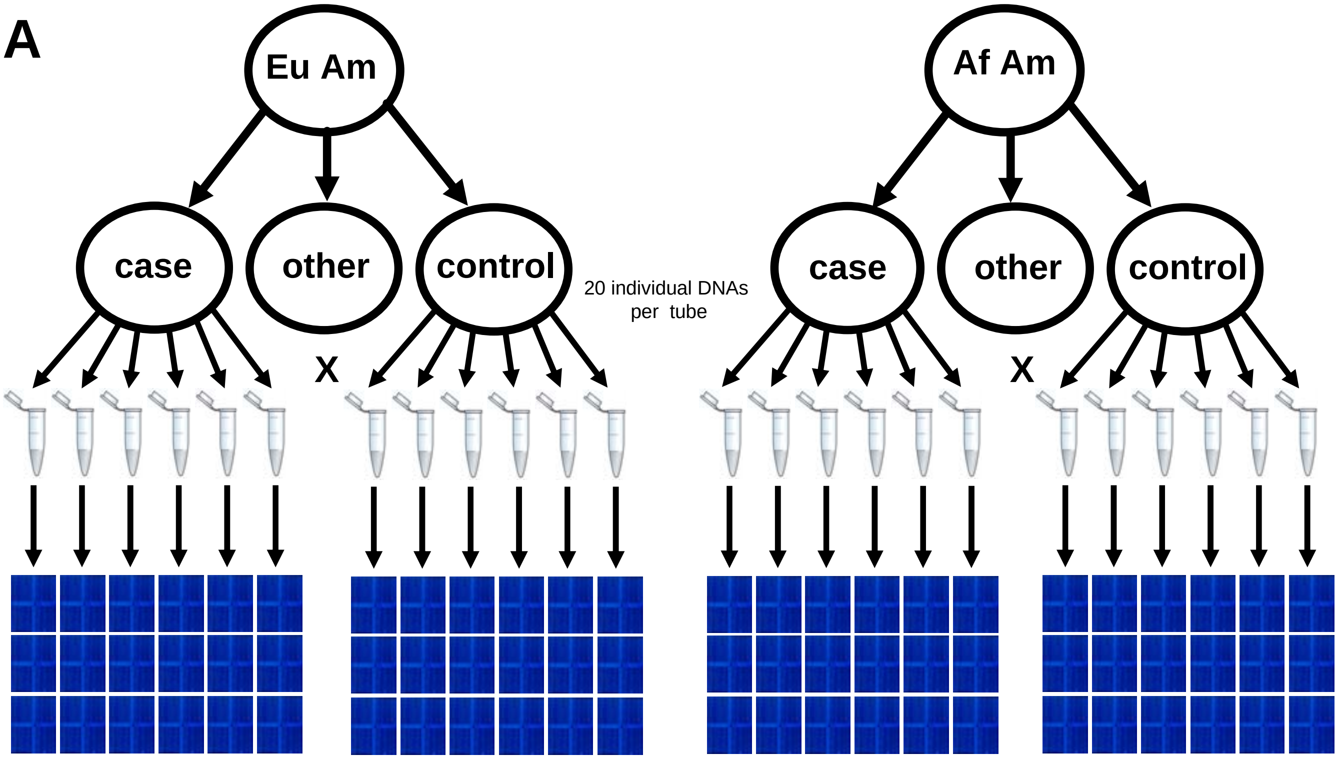

B

1) Average hybridization for each allele for each SNP for each DNA pool (normalized/background corrected, 3 arrays, 3-4 replicates/array)
2) Average arctangent of allele hybridization intensity ratio A/B for each SNP for each DNA pool
3) p values (t test) for case *vs* control differences (each SNP, each sample)

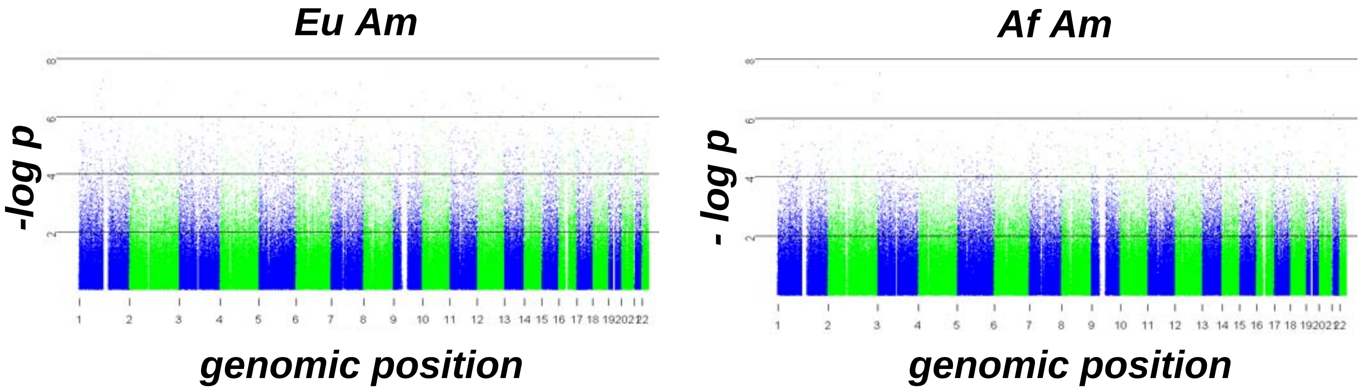

4a)Identify chromosomal regions that display “converge then cluster results” (at least 4 SNPs with p<0.05 in each sample that lie within 10 kb of each other).
4b)Identify chromosomal regions that display clustered positive results in each sample (“cluster than converge”).
5) List annotated genes within these chromosomal regions.
