# Supplementary figures and images for: Genome Wide Association for Addiction: Replicated Results and Comparisons of Two Analytic Approaches
Source: PLoS One. 2010 Jan 21;5(1):e8832. doi: 10.1371/journal.pone.0008832 (PMC2809089; doi:10.1371/journal.pone.0008832)

## QQ plots of NIDAll t dist

African American

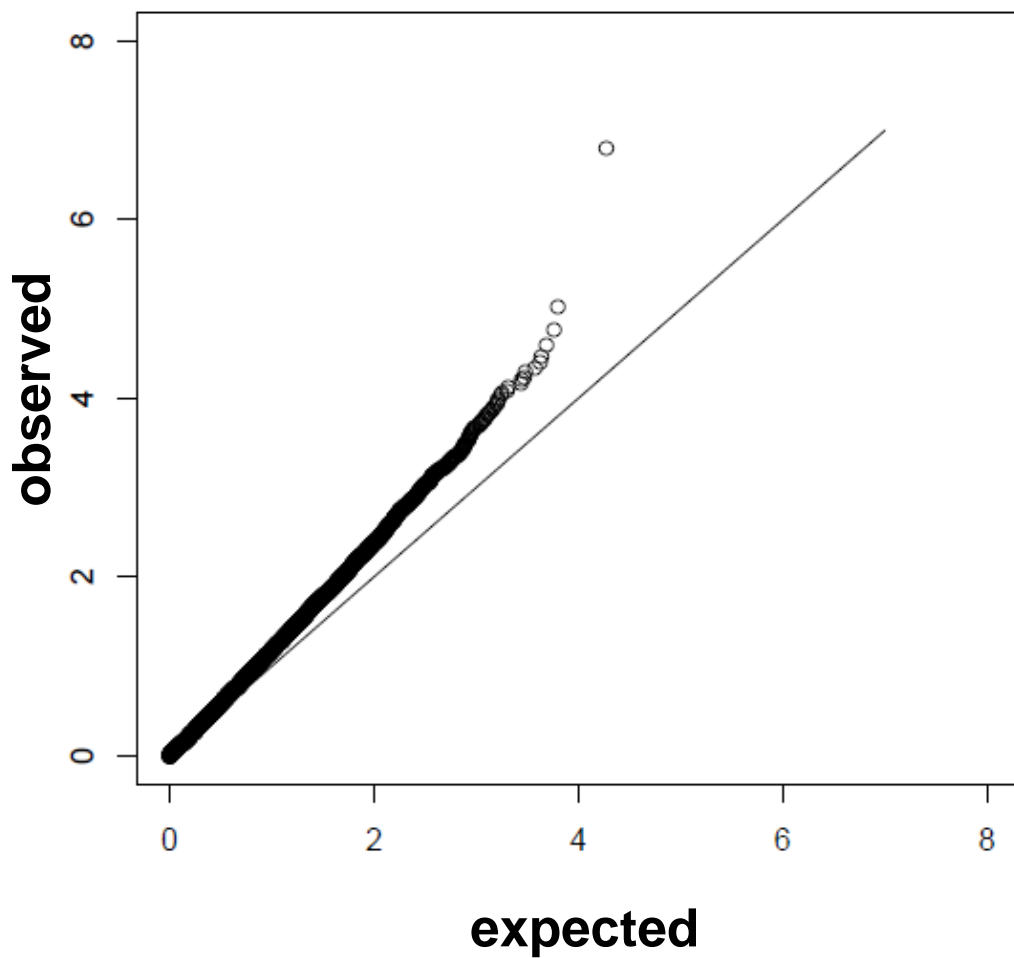

European American

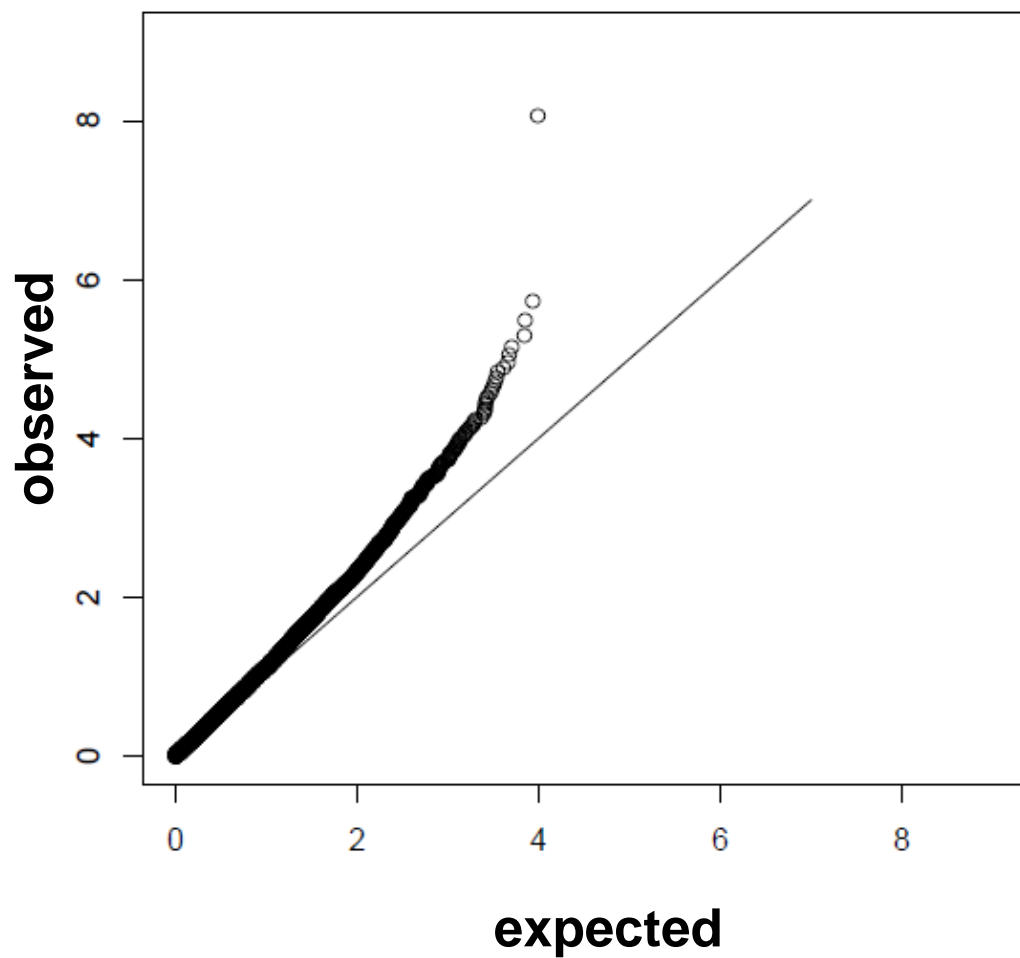

Supplement: Figure S2 — QQ plots for distribution of t values from African-American data (left) and European American data (right). Observed data from these experiments provides deviations from expected data generated from 10,000 t tests which were each run from a set of random values of the same size as those obtained from the true datasets. Deviations noted at the right side of these plots are likely to represent both a) true case vs control differences and b) nonnormal differences in the distribution of t values from the bona fide data. In the current “nontemplate” analyses, we use t values to identify the 5% of SNPs with the highest t values and subsequent testing with empirical statistics to assign overall levels of significance. Thus, any nonnormal component of this distribution is of less concern than it would be for “template” GWA analyses in which t values might be used as the primary determinant of (e.g., genome wide) significance. (0.03 MB PDF) [file pone.0008832.s002.pdf]
