# Supplementary material for: Genome Wide Association for Addiction: Replicated Results and Comparisons of Two Analytic Approaches
Source: PLoS One. 2010 Jan 21;5(1):e8832. doi: 10.1371/journal.pone.0008832 (PMC2809089; doi:10.1371/journal.pone.0008832)
Supplement: Table S1 — Genes identified by “cluster then converge” secondary analyses (2), as described in the text. These genes are thus each identified by clusters of four or more SNPs that display nominally significant allele frequency differences between polysubstance abuser vs control comparisons, cluster within <10kb of each other and lie within the gene's exons or within +/−10 kb 3′ or 5′ flanking sequences. Note that for this analysis, the same SNPs are not required to display nominal significance in each of the two samples. p values are based on 10,000 Monte Carlo simulation trials in which the number of times randomly-selected segments of the genome that lie within genes are assessed for the same features displayed by the actual gene identified. Some, but not all, of these genes are also identified by (1) converge then cluster analyses as noted in Table 1 and by clustered nominally-significant SNPs from dbGAP datasets, as noted in Table 2. (0.04 MB PDF) [file pone.0008832.s003.pdf]

Supplementary Table S1.

Gene	*chr*	*Base pair coordinate*	*Description*	*positive SNPs AA*	*positive SNPs EA*	*p-value*
RP1-21O18.1	1	14,797,800	kazrin	6	7	0.0695
GLIS1	1	53,744,494	GLIS family zinc finger 1	9	5	0.0348
PCSK9	1	55,277,808	proprotein convertase subtilisin/kexin type 9	5	4	0.0368
DAB1	1	57,236,167	disabled homolog 1 (Drosophila)	14	24	0.0262
NFIA	1	61,320,881	nuclear factor I/A	9	4	0.0655
SGIP1	1	66,772,553	SH3-domain GRB2-like (endophilin) interacting protein 1	14	6	0.0167
ST6GALNAC3	1	76,312,992	ST6 (alpha-N-acetyl-neuraminyl-2,3-beta-galactosyl-1,3)-N-acetylgalactosaminide alpha-2,6-sialyltransferase 3	16	5	0.0305
AK5	1	77,520,330	adenylate kinase 5	10	4	0.0463
KCND3	1	112,119,977	potassium voltage-gated channel, Shal-related subfamily, 3	6	10	0.0206
SPRR4	1	151,209,752	small proline rich protein 4	3	5	0.0278
DDR2	1	160,868,852	discoidin domain receptor family, member 2	5	4	0.0654
DPT	1	166,931,330	Dermatopontin	4	8	0.0229
DNM3	1	170,077,261	dynamin 3	13	7	0.0417
NR5A2	1	198,263,393	nuclear receptor subfamily 5, group A, member 2	4	4	0.0895
NFASC	1	203,064,446	neurofascin homolog (chicken)	6	4	0.0618
HHAT	1	208,568,920	hedgehog acyltransferase	5	8	0.0585
PTPN14	1	212,597,634	protein tyrosine phosphatase, non-receptor type 14	10	4	0.0285
ESRRG	1	214,743,211	estrogen-related receptor gamma	18	14	0.0121
TGFB2	1	216,586,491	transforming growth factor, beta 2	8	4	0.0299
SLC35F3	1	232,107,302	solute carrier family 35, member F3	20	4	0.0186
PLD5	1	240,318,895	phospholipase D family, member 5	20	16	0.0075
KIF26B	1	243,384,910	kinesin family member 26B	11	16	0.0152
TAF1B	2	9,901,024	TATA box binding protein (TBP)-associated factor, RNA polymerase I, B, 63kDa	4	5	0.0496
VSNL1	2	17,585,288	visinin-like 1	5	7	0.0292
GALNT14	2	30,986,837	UDP-N-acetyl-alpha-D-galactosamine:polypeptide N-acetylgalactosaminyltransferase 14 (GalNAc-T14)	5	4	0.0778
LTBP1	2	33,025,896	latent transforming growth factor beta binding protein 1	6	5	0.0855
CRIM1	2	36,436,901	cysteine rich transmembrane BMP regulator 1 (chordin-like)	5	10	0.0226
FLJ16124	2	65,517,349	FLJ16124 protein	4	4	0.2335
NPAS2	2	100,803,045	neuronal PAS domain protein 2	6	5	0.0414
SLC9A4	2	102,456,194	solute carrier family 9 (sodium/hydrogen exchanger), member 4	11	6	0.0106
ACOXL	2	111,273,085	acyl-Coenzyme A oxidase-like	5	4	0.0853
CNTNAP5	2	124,499,334	contactin associated protein-like 5	5	6	0.1750
IWS1	2	127,954,860	IWS1 homolog (S. cerevisiae)	3	6	0.0311
NAP5	2	133,398,960	Nck-associated protein 5	9	5	0.1008
LRP1B	2	140,705,466	low density lipoprotein-related protein 1B (deleted in tumors)	41	4	0.0231
LRP2	2	169,693,106	low density lipoprotein-related protein 2	4	5	0.0833
TTC30A	2	178,187,272	tetratricopeptide repeat domain 30A	5	5	0.0204
PDE11A	2	178,201,057	phosphodiesterase 11A	3	19	0.0151
ZNF533	2	180,014,954	zinc finger protein 533	15	4	0.0318
PARD3B	2	205,118,761	par-3 partitioning defective 3 homolog B	11	5	0.1295
ERBB4	2	211,955,060	v-erb-a erythroblastic leukemia viral oncogene homolog 4 (avian)	9	5	0.1970
XRCC5	2	216,682,378	X-ray repair complementing defective repair in Chinese hamster cells 5 (double-strand-break rejoining; Ku autoantigen, 80kDa)	6	4	0.0422
PID1	2	229,596,934	phosphotyrosine interaction domain containing 1	4	6	0.0675

DNER	2	229,930,592	delta-notch-like EGF repeat-containing transmembrane	5	7	0.0635
CHL1	3	213,650	cell adhesion molec. homologous to L1CAM (close homolog of L1)	8	12	0.0124
CNTN4	3	2,117,247	contactin 4	21	15	0.0197
ITPR1	3	4,510,137	inositol 1,4,5-triphosphate receptor, type 1	6	5	0.0670
THRB	3	24,134,709	thyroid hormone receptor, beta (erythroblastic leukemia viral (v-erb-a) oncogene homolog 2, avian)	6	4	0.0985
RBMS3	3	29,297,947	RNA binding motif, single stranded interacting protein	15	13	0.0273
FYCO1	3	45,934,400	FYVE and coiled-coil domain containing 1	4	5	0.0487
CACNA2D3	3	54,131,733	calcium channel, voltage-dependent, alpha 2/delta 3 subunit	14	18	0.0305
ERC2	3	55,517,376	ELKS/RAB6-interacting/CAST family member 2	8	5	0.1595
FAM3D	3	58,594,710	family with sequence similarity 3, member D	5	4	0.0398
CADPS	3	62,359,061	Ca2+-dependent secretion activator	6	4	0.1190
MAGI1	3	65,314,946	membrane associated guanylate kinase, WW and PDZ domain containing 1	5	9	0.0975
LRIG1	3	66,511,911	leucine-rich repeats and immunoglobulin-like domains 1	6	10	0.0160
FRMD4B	3	69,300,623	FERM domain containing 4B	6	4	0.0715
KALRN	3	125,296,275	kalirin, RhoGEF kinase	12	8	0.0466
UROC1	3	127,682,814	urocanase domain containing 1	4	4	0.0575
EPHB1	3	135,996,950	EPH receptor B1	4	5	0.1385
SCHIP1	3	160,474,238	schwannomin interacting protein 1	4	6	0.1410
MDS1	3	170,349,580	myelodysplasia syndrome 1	5	4	0.1370
NAALADL2	3	176,059,805	N-acetylated alpha-linked acidic dipeptidase-like 2	17	5	0.0625
DGKG	3	187,349,706	diacylglycerol kinase, gamma 90kDa	4	4	0.1080
STK32B	4	5,104,428	serine/threonine kinase 32B	8	25	0.0047
SORCS2	4	7,245,373	sortilin-related VPS10 domain containing receptor 2	9	15	0.0207
HS3ST1	4	11,009,086	heparan sulfate (glucosamine) 3-O-sulfotransferase 1	6	4	0.0289
PROM1	4	15,578,955	prominin 1	4	9	0.0244
LDB2	4	16,112,262	LIM domain binding 2	6	4	0.1000
KCNIP4	4	20,339,337	Kv channel interacting protein 4	9	9	0.0878
GPR125	4	21,998,097	G protein-coupled receptor 125	4	5	0.0623
BTC	4	75,890,472	Betacellulin	4	5	0.0450
DKFZP564O0823	4	76,077,322	DKFZP564O0823 protein	5	6	0.0288
UNC5C	4	96,308,712	unc-5 homolog C (C. elegans)	14	4	0.0311
SYNPO2	4	120,029,444	synaptopodin 2	24	6	0.0033
TBC1D9	4	141,761,387	TBC1 domain family, member 9 (with GRAM domain)	4	9	0.0264
IRF2	4	185,545,909	interferon regulatory factor 2	4	11	0.0194
ADAMTS16	5	5,193,581	ADAM metallopeptidase with thrombospondin type 1 motif, 16	11	4	0.0228
ADCY2	5	7,449,345	adenylate cyclase 2 (brain)	16	6	0.0192
MYO10	5	16,718,413	myosin X	4	15	0.0234
CDH6	5	31,229,553	cadherin 6, type 2, K-cadherin (fetal kidney)	4	15	0.0109
ARL15	5	53,216,371	ADP-ribosylation factor-like 15	5	4	0.1305
PDE4D	5	58,302,468	phosphodiesterase 4D, cAMP-specific (phosphodiesterase E3 dunce homolog, Drosophila)	10	4	0.0885
RGNEF	5	72,957,739	Rho-guanine nucleotide exchange factor	5	7	0.0570
ARSB	5	78,108,788	arylsulfatase B	4	10	0.0338
FBXL17	5	107,223,348	F-box and leucine-rich repeat protein 17	10	6	0.0557
CAMK4	5	110,587,981	calcium/calmodulin-dependent protein kinase IV	5	4	0.0865
MCC	5	112,389,428	mutated in colorectal cancers	15	8	0.0101
SEMA6A	5	115,807,150	sema domain, transmembrane domain (TM), and cytoplasmic domain, (semaphorin) 6A	4	4	0.0863
HMHB1	5	143,171,919	histocompatibility (minor) HB-1	4	6	0.0219
DPYSL3	5	146,750,564	dihydropyrimidinase-like 3	6	4	0.0346

SCGB3A2	5	147,238,467	secretoglobin, family 3A, member 2	3	5	0.0314
MGC23985	5	147,252,464	similar to AVLV472	8	7	0.0075
SLIT3	5	168,025,857	slit homolog 3 (Drosophila)	16	4	0.0513
DOCK2	5	168,996,871	dedicator of cytokinesis 2	14	7	0.0239
ADAMTS2	5	178,473,474	ADAM metallopeptidase with thrombospondin type 1 motif, 2	9	4	0.0459
NEDD9	6	11,291,519	neural precursor cell expressed, developmentally down-regulated 9	5	4	0.0411
GFOD1	6	13,471,798	glucose-fructose oxidoreductase domain containing 1	9	4	0.0270
ATXN1	6	16,407,322	ataxin 1	4	17	0.0240
LRFN2	6	40,467,351	leucine rich repeat and fibronectin type III domain containing 2	4	5	0.0800
CLIC5	6	45,974,171	chloride intracellular channel 5	10	4	0.0242
HMGCLL1	6	55,407,132	3-hydroxymethyl-3-methylglutaryl-Coenzyme A lyase-like 1	4	12	0.0187
COL9A1	6	70,982,529	collagen, type IX, alpha 1	7	5	0.0254
UBE2J1	6	90,093,063	ubiquitin-conjugating enzyme E2, J1 (UBC6 homolog, yeast)	3	3	0.0554
RRAGD	6	90,134,313	Ras-related GTP binding D	4	4	0.0601
BACH2	6	90,692,969	BTB and CNC homology 1, basic leucine zipper transcripti. factor 2	3	4	0.1485
SLC22A16	6	110,852,599	solute carrier family 22 (organic cation transporter), member 16	4	5	0.0439
NKAIN2	6	124,166,768	Na+/K+ transporting ATPase interacting 2	5	4	0.2800
PTPRK	6	128,331,625	protein tyrosine phosphatase, receptor type, K	7	8	0.0755
MYB	6	135,544,146	v-myb myeloblastosis viral oncogene homolog (avian)	4	5	0.0383
PDE7B	6	136,214,527	phosphodiesterase 7B	5	11	0.0316
PARK2	6	161,689,661	Parkinson disease (autosomal recessive, juvenile) 2, parkin	14	13	0.0860
PACRG	6	163,068,154	PARK2 co-regulated	9	4	0.1055
RPS6KA2	6	166,742,844	ribosomal protein S6 kinase, 90kDa, polypeptide 2	4	18	0.0196
ICA1	7	8,119,339	islet cell autoantigen 1, 69kDa	4	4	0.0878
THSD7A	7	11,380,787	thrombospondin, type I, domain containing 7A	14	10	0.0171
HDAC9	7	18,501,894	histone deacetylase 9	6	4	0.1300
DNAH11	7	21,549,358	dynein, axonemal, heavy polypeptide 11	14	4	0.0310
CREB5	7	28,305,465	cAMP responsive element binding protein 5	18	4	0.0243
CHN2	7	29,200,646	chimerin (chimaerin) 2	5	5	0.0725
C7orf16	7	31,693,372	chromosome 7 open reading frame 16	4	6	0.0259
AOAH	7	36,519,134	acyloxyacyl hydrolase (neutrophil)	4	8	0.0437
POU6F2	7	39,012,933	POU domain, class 6, transcription factor 2	10	14	0.0186
HECW1	7	43,118,723	HECT, C2 and WW domain containing E3 ubiquitin protein ligase 1	10	8	0.0348
DDC	7	50,493,628	dopa decarboxylase (aromatic L-amino acid decarboxylase)	4	16	0.0101
MAGI2	7	77,484,310	membrane associated guanylate kinase, WW and PDZ domain containing 2	45	16	0.0044
RELN	7	102,899,473	reelin	9	5	0.0718
TRB@	7	141,645,314	T cell receptor beta locus	4	9	0.0873
DPP6	7	154,060,464	dipeptidyl-peptidase 6	4	4	0.1110
PTPRN2	7	157,024,516	protein tyrosine phosphatase, receptor type, N polypeptide 2	4	8	0.2085
FBXO25	8	346,808	F-box protein 25	4	4	0.0650
DLGAP2	8	1,436,976	discs, large (Drosophila) homolog-associated protein 2	4	4	0.0995
MCPH1	8	6,276,480	microcephaly, primary autosomal recessive 1	13	11	0.0089
DLC1	8	12,985,243	deleted in liver cancer 1	10	9	0.0306
SGCZ	8	13,991,744	sarcoglycan zeta	16	28	0.0136
MTMR7	8	17,199,910	myotubularin related protein 7	4	7	0.0350
MTUS1	8	17,545,583	mitochondrial tumor suppressor 1	4	4	0.0935
PSD3	8	18,432,343	pleckstrin and Sec7 domain containing 3	4	16	0.0375
ADCY8	8	131,861,729	adenylate cyclase 8 (brain)	6	5	0.0536
TG	8	133,948,387	thyroglobulin	4	4	0.1185
ZNF406	8	135,559,213	zinc finger protein 406	6	4	0.0678

GLIS3	9	3,817,676	GLIS family zinc finger 3	13	18	0.0064
JMJD2C	9	6,748,083	jumonji domain containing 2C	22	14	0.0071
PTPRD	9	8,307,268	protein tyrosine phosphatase, receptor type, D	11	11	0.0421
C9orf39	9	17,252,114	chromosome 9 open reading frame 39	7	4	0.0621
C9orf138	9	18,917,893	chromosome 9 open reading frame 138	10	6	0.0178
LRRN6C	9	27,938,528	leucine rich repeat neuronal 6C	28	20	0.0182
PIP5K1B	9	70,510,436	phosphatidylinositol-4-phosphate 5-kinase, type I, beta	5	18	0.0118
RORB	9	76,302,072	RAR-related orphan receptor B	10	6	0.0260
ROR2	9	93,524,705	receptor tyrosine kinase-like orphan receptor 2	6	4	0.0670
HSD17B3	9	98,037,410	hydroxysteroid (17-beta) dehydrogenase 3	4	5	0.0481
GABBR2	9	100,090,187	gamma-aminobutyric acid (GABA) B receptor, 2	13	6	0.0283
ABCA1	9	106,583,104	ATP-binding cassette, sub-family A (ABC1), member 1	3	6	0.0555
PALM2-AKAP2	9	111,582,410	PALM2-AKAP2 protein	8	10	0.0344
ASTN2	9	118,227,328	astrotactin 2	6	14	0.0773
PTGS1	9	124,173,050	prostaglandin-endoperoxide synthase 1 (prostaglandin G/H synthase and cyclooxygenase)	5	5	0.0243
ABL1	9	132,579,089	v-abl Abelson murine leukemia viral oncogene homolog 1	4	10	0.0289
VAV2	9	135,616,837	vav 2 oncogene	5	4	0.0790
EHMT1	9	139,725,238	euchromatic histone-lysine N-methyltransferase 1	8	4	0.0353
DIP2C	10	311,432	DIP2 disco-interacting protein 2 homolog C (Drosophila)	5	4	0.1220
PFKP	10	3,099,752	phosphofructokinase, platelet	4	4	0.0710
CUGBP2	10	11,087,290	CUG triplet repeat, RNA binding protein 2	14	4	0.0284
CUBN	10	16,906,534	cubilin (intrinsic factor-cobalamin receptor)	11	4	0.0418
CACNB2	10	18,469,612	calcium channel, voltage-dependent, beta 2 subunit	8	4	0.0828
C10orf112	10	19,564,058	chromosome 10 open reading frame 112	4	9	0.0768
PLXDC2	10	20,145,378	plexin domain containing 2	8	4	0.0985
KIAA1217	10	24,538,099	KIAA1217	6	4	0.0815
GDF10	10	48,045,795	growth differentiation factor 10	4	5	0.0315
PRKG1	10	52,504,299	protein kinase, cGMP-dependent, type I	21	10	0.0498
ADAMTS14	10	72,102,565	ADAM metallopeptidase with thrombospondin type 1 motif, 14	13	5	0.0117
NRG3	10	83,625,077	neuregulin 3	5	4	0.3085
RGR	10	85,994,789	retinal G protein coupled receptor	3	4	0.0492
GRID1	10	87,349,292	glutamate receptor, ionotropic, delta 1	6	18	0.0344
SORBS1	10	97,061,520	sorbin and SH3 domain containing 1	11	8	0.0174
ATRNL1	10	116,843,114	attractin-like 1	10	5	0.1180
GFRA1	10	117,812,943	GDNF family receptor alpha 1	10	14	0.0067
TACC2	10	123,738,679	transforming, acidic coiled-coil containing protein 2	9	9	0.0198
CTBP2	10	126,667,391	C-terminal binding protein 2	7	20	0.0046
UROS	10	127,467,142	uroporphyrinogen III synthase (congenital erythropoietic porphyria)	4	4	0.0537
GALNTL4	11	11,248,999	UDP-N-acetyl-alpha-D-galactosamine:polypeptide N-acetylgalactosaminyltransferase-like 4	6	4	0.0888
MICAL2	11	12,088,714	microtubule associated monoxygenase, calponin and LIM domain containing 2	9	8	0.0214
NELL1	11	20,647,712	NEL-like 1 (chicken)	4	46	0.0031
UVRAG	11	75,203,923	UV radiation resistance associated gene	4	5	0.0990
MAML2	11	95,351,088	mastermind-like 2 (Drosophila)	9	7	0.0357
CNTN5	11	98,397,081	contactin 5	14	10	0.0975
COP1	11	104,417,263	caspase-1 dominant-negative inhibitor pseudo-ICE	4	3	0.0429
DSCAML1	11	116,803,699	Down syndrome cell adhesion molecule like 1	15	4	0.0273
GRIK4	11	120,036,238	glutamate receptor, ionotropic, kainate 4	12	8	0.0172
STS-1	11	122,031,640	Cbl-interacting protein Sts-1	4	10	0.0255

KIRREL3	11	125,799,613	kin of IRRE like 3 (Drosophila)	4	14	0.0458
OPCML	11	131,790,085	opioid binding protein/cell adhesion molecule-like	11	13	0.0703
TSPAN9	12	3,056,818	tetraspanin 9	4	5	0.0798
PLEKHA5	12	19,173,995	pleckstrin homology domain containing, family A member 5	5	4	0.0865
LRMP	12	25,096,508	lymphoid-restricted membrane protein	7	4	0.0300
DCD	12	53,324,642	dermcidin	5	3	0.0299
PTPRB	12	69,201,231	protein tyrosine phosphatase, receptor type, B	7	6	0.0223
CHST11	12	103,370,614	carbohydrate (chondroitin 4) sulfotransferase 11	4	4	0.1335
ACACB	12	108,061,594	acetyl-Coenzyme A carboxylase beta	5	8	0.0255
OAS2	12	111,900,657	2'-5'-oligoadenylate synthetase 2, 69/71kDa	4	5	0.0356
RBM19	12	112,744,245	RNA binding motif protein 19	5	12	0.0145
KSR2	12	116,389,387	kinase suppressor of ras 2	4	4	0.1510
HSPB8	12	118,100,978	heat shock 22kDa protein 8	5	5	0.0241
TMEM132B	12	124,377,115	transmembrane protein 132B	4	4	0.1525
TMEM132C	12	127,318,855	transmembrane protein 132C	17	13	0.0121
TMEM132D	12	128,122,224	transmembrane protein 132D	9	11	0.0728
RIMBP2	12	129,446,635	RIMS binding protein 2	5	4	0.0573
ATP8A2	13	24,941,115	ATPase, aminophospholipid transporter-like, Class I, type 8A, member 2	4	18	0.0280
FLT1	13	27,773,790	fms-related tyrosine kinase 1 (vascular endothelial growth factor/vascular permeability factor receptor)	4	4	0.1015
KIAA0774	13	28,496,748	KIAA0774	10	4	0.0743
SLC7A1	13	28,981,551	solute carrier family 7 (cationic amino acid transporter, y+ system), member 1	11	9	0.0076
TRPC4	13	37,108,795	transient receptor potential cation channel, subfamily C, member 4	4	4	0.0998
PIG38	13	42,685,704	proliferation-inducing protein 38	5	5	0.1070
FLJ32682	13	45,013,433	hypothetical protein FLJ32682	5	5	0.0303
GPC5	13	90,848,930	glypican 5	14	18	0.0653
CLYBL	13	99,056,937	citrate lyase beta like	4	4	0.1200
VGCNL1	13	100,504,131	voltage gated channel like 1	9	4	0.0560
ITGBL1	13	100,902,967	integrin, beta-like 1 (with EGF-like repeat domains)	24	9	0.0033
MYR8	13	108,046,501	myosin heavy chain Myr 8	24	8	0.0104
COL4A1	13	109,599,311	collagen, type IV, alpha 1	18	6	0.0061
COL4A2	13	109,757,632	collagen, type IV, alpha 2	20	27	0.0015
TRA@	14	21,159,897	T cell receptor alpha locus	48	7	0.0011
STXBP6	14	24,351,133	syntaxin binding protein 6 (amisyn)	4	8	0.0560
AKAP6	14	31,868,274	A kinase (PRKA) anchor protein 6	4	15	0.0342
NPAS3	14	32,478,200	neuronal PAS domain protein 3	12	4	0.0995
SLC25A21	14	36,218,829	solute carrier family 25 (mitochondrial oxodicarboxylate carrier), member 21	6	4	0.1245
PELI2	14	55,654,846	pellino homolog 2 (Drosophila)	13	11	0.0087
RTN1	14	59,132,447	reticulon 1	4	8	0.0605
KCNH5	14	62,243,698	potassium voltage-gated channel, subfamily H (eag-related), member 5	4	9	0.0633
RGS6	14	71,469,586	regulator of G-protein signalling 6	19	24	0.0058
JDP2	14	74,968,590	jun dimerization protein 2	4	4	0.0605
FLRT2	14	85,066,241	fibronectin leucine rich transmembrane protein 2	6	11	0.0137
CHES1	14	88,692,274	checkpoint suppressor 1	4	4	0.1080
SNRPN	15	22,619,887	small nuclear ribonucleoprotein polypeptide N	4	9	0.0968
RYR3	15	31,390,469	ryanodine receptor 3	12	21	0.0080
FBN1	15	46,487,797	fibrillin 1	4	4	0.1080
UNC13C	15	52,092,393	unc-13 homolog C (C. elegans)	4	7	0.1270

CGNL1	15	55,455,997	cingulin-like 1	6	11	0.0189
RORA	15	58,576,755	RAR-related orphan receptor A	14	9	0.0439
TLN2	15	60,726,802	talin 2	5	8	0.0325
SLCO3A1	15	90,197,950	solute carrier organic anion transporter family, member 3A1	6	4	0.0875
ADAMTS17	15	98,331,993	ADAM metallopeptidase with thrombospondin type 1 motif, 17	4	15	0.0279
A2BP1	16	6,009,133	ataxin 2-binding protein 1	84	42	0.0012
FTO	16	52,295,376	fatso	19	8	0.0140
KIAA1576	16	76,379,984	KIAA1576 protein	11	6	0.0156
PKD1L2	16	79,691,991	polycystic kidney disease 1-like 2	9	4	0.0292
GAN	16	79,906,076	giant axonal neuropathy (gigaxonin)	5	8	0.0167
KIAA1609	16	83,068,608	KIAA1609	6	5	0.0203
OR3A4	17	3,160,355	olfactory receptor, family 3, subfamily A, member 4	4	4	0.0422
TRPV1	17	3,415,491	transient receptor potential cation channel, subfamily V, member 1	5	4	0.0405
FLJ45455	17	11,085,771	FLJ45455 protein	6	10	0.0274
ACCN1	17	28,364,218	amiloride-sensitive cation channel 1, neuronal (degenerin)	6	5	0.0565
MSI2	17	52,688,930	musashi homolog 2 (Drosophila)	5	5	0.0883
PRKCA	17	61,729,388	protein kinase C, alpha	5	4	0.1405
SLC39A11	17	68,153,683	solute carrier family 39 (metal ion transporter), member 11	4	6	0.1075
PTPRM	18	7,557,817	protein tyrosine phosphatase, receptor type, M	5	7	0.1455
CDH2	18	23,784,933	cadherin 2, type 1, N-cadherin (neuronal)	5	5	0.0595
C18orf24	18	46,155,390	chromosome 18 open reading frame 24	5	5	0.0241
NEDD4L	18	53,862,778	neural precursor cell expressed, developmentally down-regulated 4-like	8	9	0.0324
CCBE1	18	55,252,124	collagen and calcium binding EGF domains 1	4	10	0.0419
NETO1	18	68,565,767	neuropilin (NRP) and tolloid (TLL)-like 1	10	5	0.0212
FAUP1	18	70,208,099	FBR-MuSV-associated ubiquitously expressed (fox derived) pseudogene 1	4	4	0.0360
ZNF407	18	70,474,282	zinc finger protein 407	3	4	0.1220
TGM6	20	2,309,554	transglutaminase 6	3	5	0.0434
PLCB1	20	8,061,296	phospholipase C, beta 1 (phosphoinositide-specific)	15	4	0.0698
OTOR	20	16,677,003	otoraplin	4	4	0.0388
PCSK2	20	17,155,631	proprotein convertase subtilisin/kexin type 2	4	4	0.1095
BFSP1	20	17,422,550	beaded filament structural protein 1, filensin	5	4	0.0383
C20orf26	20	19,981,196	chromosome 20 open reading frame 26	10	10	0.0232
PTPRT	20	40,134,806	protein tyrosine phosphatase, receptor type, T	27	15	0.0140
C20orf119	20	42,972,117	chromosome 20 open reading frame 119	5	5	0.0258
EYA2	20	44,956,916	eyes absent homolog 2 (Drosophila)	4	5	0.0890
BCAS4	20	48,844,874	breast carcinoma amplified sequence 4	4	4	0.0653
TSHZ2	20	51,022,353	teashirt family zinc finger 2	15	6	0.0302
VAPB	20	56,397,651	VAMP (vesicle-associated membrane protein)-associated protein B and C	4	11	0.0149
PHACTR3	20	57,612,998	phosphatase and actin regulator 3	5	10	0.0253
APP	21	26,174,732	amyloid beta (A4) precursor protein (peptidase nexin-II, Alzheimer disease)	12	3	0.0302
GRIK1	21	29,831,125	glutamate receptor, ionotropic, kainate 1	11	12	0.0208
TIAM1	21	31,414,352	T-cell lymphoma invasion and metastasis 1	8	9	0.0430
KCNJ6	21	37,918,655	potassium inwardly-rectifying channel, subfamily J, member 6	4	8	0.0580
MX1	21	41,720,024	myxovirus (influenza virus) resistance 1, interferon-inducible protein p78 (mouse)	3	3	0.0579
TMPRSS2	21	41,758,351	transmembrane protease, serine 2	4	4	0.0587
C21orf25	21	42,178,290	chromosome 21 open reading frame 25	4	4	0.0545
C21orf29	21	44,742,203	chromosome 21 open reading frame 29	5	5	0.0565

DIP2A	21	46,703,318	DIP2 disco-interacting protein 2 homolog A (Drosophila)	6	5	0.0308
IGL@	22	20,710,659	immunoglobulin lambda locus	5	4	0.2380
MYO18B	22	24,468,120	myosin XVIIIB	17	8	0.0090
SEZ6L	22	24,895,480	seizure related 6 homolog (mouse)-like	18	4	0.0106
MRPS18CP6	22	43,258,029	mitochondrial ribosomal protein S18C pseudogene 6	3	3	0.0443
PRR5	22	43,443,257	proline rich 5 (renal)	4	5	0.0429
