# Supplementary material for: Genome Wide Association for Addiction: Replicated Results and Comparisons of Two Analytic Approaches
Source: PLoS One. 2010 Jan 21;5(1):e8832. doi: 10.1371/journal.pone.0008832 (PMC2809089; doi:10.1371/journal.pone.0008832)
Supplement: Table S2 — List of RS numbers and chromosomal location identified as “positive” that are the basis for identification of the genes in Table 2 in the manuscript. (0.03 MB PDF) [file pone.0008832.s004.pdf]

rs_id	chr	pos
rs2236860	1	29013335
rs678849	1	29017775
rs4654322	1	29023832
rs419335	1	29024431
rs2236856	1	29034412
rs2236855	1	29034586
rs760589	1	29035052
rs760588	1	29035155
rs538485	1	29037148
rs10753331	1	29037169
rs7522397	1	46799176
rs3766239	1	46808663
rs3766238	1	46808902
rs5015171	1	49080600
rs2355689	1	49093419
rs1934386	1	49096915
rs2153326	1	49097877
rs10493137	1	49098403
rs6684570	1	49103391
rs10888631	1	49104053
rs6667780	1	49107917
rs9660009	1	49108184
rs10788902	1	49191902
rs11205576	1	49193417
rs12097797	1	49200890
rs6669448	1	49201071
rs7527167	1	49202012
rs1286825	1	61999576
rs11587744	1	62018212
rs11207833	1	62020955
rs12069192	1	93815435
rs7546785	1	93819250
rs236327	1	93830554
rs17160062	1	145197991
rs11800580	1	145199331
rs17360443	1	145218034
rs1780341	1	162943886
rs12026184	1	162952600
rs2200797	1	162962818
rs16834333	1	162983471
rs6703951	1	162994836
rs12731646	1	167357284
rs3766039	1	167357336
rs10919065	1	167360181
rs7517394	1	167360386
rs968304	1	167360724
rs12086569	1	167377464
rs12691502	1	167379255
rs12354315	1	167391263
rs1419323	1	167392419
rs3766053	1	167393025

rs12744184	1	167395775
rs12755385	1	167398174
rs12562404	1	167398858
rs12074013	1	167400245
rs10919079	1	167400429
rs10800408	1	167403457
rs7541564	1	167406175
rs3766060	1	167411858
rs10919086	1	167415007
rs3820055	1	167415883
rs3820056	1	167415902
rs4656658	1	167416877
rs12067018	1	167416957
rs3766064	1	167424543
rs909931	1	167429202
rs909930	1	167429227
rs2040446	1	167431890
rs1018832	1	167432117
rs3753297	1	167443401
rs36040633	1	167447551
rs10800418	1	167450009
rs6688038	1	167457196
rs12145969	1	167458734
rs6703463	1	167466049
rs10732287	1	167543440
rs1856303	1	167547862
rs12073439	1	167551106
rs7533257	1	167553784
rs10800432	1	167559464
rs16855388	1	178416801
rs3767179	1	178418883
rs12145255	1	178435990
rs6548131	2	3209395
rs6548139	2	3221041
rs6548144	2	3221865
rs1869417	2	3228940
rs1869416	2	3229255
rs13419506	2	3237329
rs10198129	2	3241821
rs7591840	2	3244741
rs12621297	2	3245221
rs6752993	2	3246172
rs2602765	2	3248172
rs4668453	2	15644099
rs4668940	2	15667150
rs976016	2	15690316
rs3820732	2	46198473
rs17034576	2	46199790
rs6719520	2	46200318
rs6719933	2	99630406
rs10201444	2	99648153
rs17022868	2	99657711

rs3792167	2	160553828
rs4664305	2	160567481
rs3792174	2	160570960
rs1355418	2	160571455
rs6432836	2	166123345
rs16850912	2	166129660
rs16850913	2	166130009
rs16850914	2	166130256
rs6719076	2	166351525
rs4340537	2	166356917
rs13024739	2	166360610
rs16851058	2	166367988
rs7605770	2	169137081
rs2077765	2	169156318
rs2077764	2	169156339
rs7588907	2	185443844
rs7564941	2	185444058
rs11904676	2	185458965
rs12693400	2	185466657
rs1366840	2	185476099
rs17431742	2	185481646
rs10427295	2	185504662
rs6726421	2	185505087
rs6755404	2	185505473
rs17509873	2	185505653
rs1874981	3	7147721
rs7650218	3	7166923
rs7621420	3	7171728
rs7645978	3	7172715
rs1818033	3	7177533
rs1499142	3	7178151
rs3804836	3	7701861
rs162784	3	7706411
rs162783	3	7706869
SNP_A-2114706	3	7714950
rs13325718	3	21468102
rs3913941	3	21468206
rs17273488	3	21473653
rs431315	3	21474549
rs7644742	3	21709238
rs7628221	3	21711307
rs12632528	3	21717029
rs2336038	3	21720044
rs6787257	3	21720640
rs17009349	3	21723793
rs957590	3	21727159
rs9875169	3	21729732
rs4858348	3	21730685
rs11712782	3	21730997
rs12106696	3	21731557
rs13077624	3	21737614
rs17009397	3	21753063

rs11717325	3	45095516
rs17634308	3	45107091
rs6800538	3	45114323
rs4343583	3	59700891
rs2121793	3	59701635
rs17061153	3	59707588
rs812965	3	59711339
rs1385717	3	59711554
rs1485723	3	59715994
rs6785349	3	59721569
rs7349527	3	59875260
rs9311741	3	59882369
rs13070872	3	59884355
rs1038516	3	59892968
rs3772447	3	59896687
rs3772448	3	59897057
rs3772452	3	59897849
rs17061716	3	59957376
rs7649040	3	59961306
rs17254765	3	59961337
rs802777	3	59966903
rs17061766	3	59971875
rs17255497	3	59972152
rs9311745	3	59976865
rs3912492	3	59977495
rs6804301	3	59978690
rs9861525	3	59979948
rs12632711	3	59982671
rs3912494	3	59982697
rs11916155	3	59984291
rs9822178	3	59984848
rs17061847	3	59987390
rs9874888	3	59991357
rs17061880	3	59993366
rs7433793	3	59994590
rs212062	3	59997510
rs212060	3	59999269
rs3845972	3	60002874
rs212057	3	60003631
rs17061923	3	60006558
rs212041	3	60013318
rs3912495	3	60014051
rs17062579	3	60199051
rs6776604	3	60199742
SNP_A-1837347	3	60200552
rs17062584	3	60201294
rs6787887	3	60325011
rs2736823	3	60334358
rs2687165	3	60340493
rs17062890	3	60341727
rs17062892	3	60342332
rs10470623	3	60351480

rs9835300	3	60353172
rs12638023	3	60556846
rs2856028	3	60563667
rs6775612	3	60564378
rs939497	3	60571383
rs939498	3	60571739
rs2856036	3	60572503
rs2856044	3	60583127
rs1009947	3	60583757
rs2885865	3	60585381
rs2363670	3	60586653
rs7633853	3	60588699
rs12637393	3	60595324
rs7633325	3	60596124
rs17063870	3	60686800
rs9865712	3	60694988
rs9990356	3	60695965
rs12493219	3	60700643
rs6785140	3	60825221
rs6785095	3	60831169
rs1374916	3	60831983
rs9834435	3	60838816
rs6780515	3	60846454
SNP_A-4223459	3	60894434
rs1868227	3	60895058
rs1868225	3	60895184
rs4974251	3	60896747
rs9827976	3	60898995
rs9990051	3	60907660
rs1562522	3	60908974
rs9849709	3	60912197
rs9868796	3	60920816
rs9881064	3	61034471
rs7650309	3	61039590
rs1439008	3	61042027
rs2083264	3	61049269
rs6764497	3	61050194
rs1347852	3	61055822
rs340142	3	113263361
rs16859145	3	113277525
rs6765246	3	113278165
rs2284994	3	186411420
rs16859819	3	186411997
rs11715222	3	186414496
rs1558379	4	17281982
rs4235389	4	17305378
rs758482	4	17312732
rs12506103	4	22298948
rs7695833	4	22306884
rs9991992	4	22308737
rs16872998	4	22313071
rs16873004	4	22314472

rs16873006	4	22315092
rs12508070	4	22315249
rs7673025	4	22356471
rs1385760	4	22379397
SNP_A-2209389	4	22386598
rs7683275	4	41221779
rs4861118	4	41223320
rs6447088	4	41235876
rs35406	5	33981900
rs35392	5	33992668
rs3756462	5	34005763
rs840380	5	34023811
rs16878635	5	71783204
rs6862221	5	71790718
rs1019457	5	71798910
rs4703908	5	71802603
rs10061668	5	71807102
rs4703910	5	71807866
rs7721377	5	71827595
rs11743229	5	71828943
rs10065019	5	71830325
rs10063693	5	71831836
rs4703937	5	71833607
rs246571	5	71840906
rs417769	5	71847234
rs17165251	5	126422383
rs17165249	5	126423410
rs17165248	5	126424037
rs10519920	5	126430778
rs17165234	5	126431534
rs12655006	5	126435501
rs10038760	5	134924732
rs2547	5	134935451
rs17168672	5	134941555
rs17168680	5	134945181
SNP_A-2247550	5	134950527
rs17168689	5	134951339
rs17168691	5	134951398
rs1148364	5	134952861
rs9284974	5	166806569
rs1030668	5	166809005
rs1019930	5	166813129
rs4869044	5	166815334
rs12656490	5	166907173
rs888977	5	166909858
rs6898357	5	166918887
rs10051918	5	166919405
rs875705	5	166921468
rs35024466	5	166922091
rs203307	5	167010028
rs10516035	5	167019479
rs1459070	5	167027799

rs1459068	5	167027981
rs17068955	5	167032842
rs1459073	5	167033284
rs17510007	5	167036730
rs17525725	5	167226230
rs1035373	5	167229006
rs1592800	5	167235817
rs12517071	5	167240564
rs1421986	5	167242922
rs1421985	5	167243005
rs1421980	5	167246839
rs11953311	5	167246983
rs11134476	5	167247118
rs11134477	5	167247158
rs2161422	5	167361210
rs12153623	5	167370293
rs17069617	5	167376943
rs17069636	5	167381635
rs6882264	5	167387579
rs247989	5	167396935
rs10516041	5	167405469
rs17069726	5	167413716
rs12657034	5	167415563
rs10080048	5	167423906
rs2337119	5	167469063
rs4976570	5	167470097
rs10073793	5	167475166
rs2617972	5	167476408
rs16891022	6	38844662
rs1738260	6	38854241
rs2038201	6	38854708
rs6940955	6	38856738
rs4714188	6	38900142
rs9380782	6	38902890
rs9357283	6	38908142
rs9296264	6	38908879
rs9380785	6	38913537
rs9380789	6	38930883
rs1280044	6	76579322
rs12212499	6	76581593
rs13194998	6	76585158
rs10943292	6	76590201
rs13216921	6	76640907
rs10498904	6	76642917
rs3798434	6	76645282
rs3798433	6	76645504
rs3798425	6	76664270
rs2749055	6	102076023
rs17816480	6	102076709
rs2749052	6	102077237
rs2518253	6	102084201
rs17829435	6	102160323

rs581633	6	102163163
rs581567	6	102163218
rs9404125	6	102163517
rs478344	6	102164131
rs2852584	6	102183608
SNP_A-1957101	6	119417042
rs659411	6	119428855
rs583635	6	119432880
rs17060099	6	131544321
rs3850237	6	131553894
rs3903759	6	131562687
rs9492861	6	131566311
rs11966670	6	131566941
rs3777473	6	131567676
rs17573187	6	131576402
rs17060121	6	131580353
rs9483230	6	131584230
rs2813484	6	152505595
rs2813485	6	152508115
rs9371241	6	152510000
rs2747665	6	152510136
rs2763015	6	152549890
rs2813524	6	152552650
rs2635458	6	152553011
rs2635457	6	152553471
rs2635462	6	152558808
rs17194424	6	152563124
rs2253512	6	152564619
rs2253407	6	152565243
rs9479263	6	152573503
rs1203233	6	152756299
rs550685	6	152760184
rs522437	6	152760911
rs544863	6	152770507
rs214987	6	152775286
rs7756410	6	152830890
rs9371600	6	152832226
rs9371601	6	152832266
rs7747960	6	152833167
rs9397510	6	152838758
rs7763880	6	152843990
rs9397512	6	152847582
rs9371603	6	152852323
rs9397518	6	152852414
rs6557233	6	152862981
rs9371604	6	152867915
rs2623971	6	152872760
rs2449116	6	152879814
rs7745725	6	152893977
rs1527369	6	152907159
rs818450	6	152930167
rs9371870	6	155550934

rs7741028	6	155569749
rs9384296	6	155572536
SNP_A-4236469	6	166694451
rs6456079	6	166702133
rs3757195	6	166719012
rs10950932	7	22980210
rs2041359	7	23001520
rs13225524	7	23004549
rs17369747	7	23004926
rs10225133	7	23006993
rs13225964	7	23007945
rs10232446	7	23014263
rs11767869	7	23016813
rs10950934	7	23023549
rs12700406	7	23027633
rs1035028	7	31833575
rs3801350	7	31838385
rs12701145	7	31850646
rs17139929	7	116904999
rs17545644	7	116922448
rs17132549	7	116929712
rs17145022	7	122273219
rs11981227	7	122292650
rs17611963	7	122307579
rs17166813	7	132284338
rs4731921	7	132286163
rs10254275	7	132294301
rs10238727	7	132294404
rs7804370	7	132295762
rs10250029	7	132302209
rs6971707	7	132304795
rs7787607	7	132308220
rs2058945	7	132308624
rs7803008	7	132321853
rs2721291	8	2785741
rs637954	8	2795672
rs1979509	8	2799115
rs10111942	8	2802591
rs10103675	8	2951910
rs750318	8	2954259
rs17390567	8	2955771
rs1841352	8	2956463
rs2045638	8	2963312
rs2125953	8	2966156
rs17079488	8	2973981
rs17390797	8	2974287
rs12542249	8	2977814
rs10046671	8	2983557
rs12545633	8	2987869
rs7818108	8	3035516
rs983778	8	3038575
rs17079576	8	3039154

rs17079607	8	3042040
rs11989577	8	3056359
rs17079637	8	3062413
rs3943652	8	3071623
rs13258433	8	3073913
rs1442403	8	3075216
rs12541333	8	3076988
rs1562116	8	3078491
rs1442408	8	3079782
rs1442410	8	3079979
rs1020347	8	3088991
rs4875412	8	3090554
rs17319596	8	3092001
rs7018325	8	3280551
rs6984069	8	3281236
rs6558769	8	3283553
rs6982744	8	3284128
rs10282982	8	3292439
rs7004179	8	3320429
rs7464308	8	3320880
rs7461879	8	3320917
rs4268145	8	3321148
rs13251344	8	3322606
rs11785476	8	3327152
rs11783144	8	3327306
rs13276523	8	3328869
rs17066293	8	3337785
rs2100119	8	3343653
rs4487801	8	3344610
rs4471061	8	3344663
rs6558786	8	3345017
rs4602914	8	3345841
rs6980585	8	3423510
rs17395515	8	3430516
SNP_A-2297240	8	3438071
rs2469359	8	3438465
rs2469358	8	3438519
rs17066505	8	3439547
rs11985561	8	3439670
rs2469354	8	3440295
rs2449175	8	3444896
rs17128	8	3445855
rs9644345	8	3449401
rs4376516	8	3491640
rs17066739	8	3491826
rs11778221	8	3492482
rs17066769	8	3494133
rs17397421	8	3495526
rs2469403	8	3495668
rs2469400	8	3496080
rs12680010	8	3497769
rs17063131	8	3503126

rs4307367	8	3534220
rs2086532	8	3535435
rs7833222	8	3536750
rs7837233	8	3536989
rs2624070	8	3537344
rs2623595	8	3540619
rs17066939	8	3540925
rs13253574	8	3541109
rs900224	8	3549234
rs7841278	8	3549935
rs17067079	8	3551589
rs2720799	8	3565398
rs7820399	8	3565440
rs2623550	8	3566702
rs12675463	8	3572760
rs1383955	8	3573885
rs2128076	8	3575438
rs10503215	8	3576424
rs17067208	8	3576909
rs13268323	8	3581586
rs1481722	8	3589278
rs7843194	8	3592989
rs1545821	8	3593993
rs2623723	8	3594595
rs12543111	8	3601637
rs11996063	8	3606157
rs12675982	8	3608343
rs12682343	8	3608532
rs1001006	8	3621309
SNP_A-1926153	8	3626836
rs2720890	8	3630895
rs1383953	8	3630967
rs1383954	8	3631039
rs2623678	8	3635195
rs2720811	8	3635728
rs7000369	8	3635859
rs2623684	8	3638118
rs10102521	8	3650879
SNP_A-2118809	8	3651836
rs11136668	8	3652805
rs11774223	8	3653990
rs11989694	8	3658498
rs9694214	8	3663632
rs9693433	8	3664116
rs4430120	8	3665560
rs10110717	8	3667590
rs7830364	8	3669431
rs11787086	8	3670962
rs4437679	8	3671287
rs10088247	8	3671607
rs2975365	8	3707971
rs17067808	8	3712244

rs2975353	8	3722046
rs2975352	8	3722057
rs2930341	8	3726148
rs2627369	8	3727600
rs2049304	8	3735700
rs2049303	8	3735937
rs2554661	8	3776175
rs10503223	8	3778626
rs2930333	8	3782733
rs2554622	8	3782940
rs2554621	8	3782982
rs2627531	8	3783028
rs2688306	8	3783510
rs2627530	8	3783529
rs17068294	8	3817272
rs11990330	8	3817745
rs6558829	8	3818444
rs2449818	8	3828025
rs17068668	8	3884156
rs12114678	8	3885748
rs3849821	8	3892422
rs7814135	8	3895215
rs1426282	8	3907290
rs6558843	8	3909332
rs1365280	8	3909528
rs4554489	8	3909568
rs4406417	8	3909682
rs2552122	8	3915929
rs2554635	8	3924312
rs17404093	8	3933775
rs10095133	8	3934466
rs13340606	8	3940031
rs13340578	8	3940374
rs13268176	8	3947332
rs17068999	8	3948052
rs17069006	8	3948810
rs17404496	8	3948876
rs17069008	8	3949083
rs4606080	8	3953407
rs2407297	8	3954397
rs12155988	8	3963273
rs17069056	8	3971988
rs10099471	8	3975628
rs4875303	8	4028983
rs7018112	8	4030154
rs3860866	8	4039531
rs968370	8	4040455
rs968371	8	4040487
rs7815311	8	4041720
rs11989184	8	4043491
rs1647314	8	4053773
rs13249547	8	4058512

rs1700068	8	4062078
rs3849842	8	4063204
rs1647319	8	4063796
rs12674947	8	4064166
rs17069552	8	4064598
rs1700062	8	4068683
rs11992222	8	4076524
rs11993028	8	4076646
rs13272378	8	4078056
rs2202390	8	4078869
rs17069682	8	4083982
rs1714689	8	4084927
rs1504769	8	4089356
rs17069759	8	4100361
rs9693235	8	4109411
rs12056402	8	4114888
rs809649	8	4118376
rs11136715	8	4118520
rs13258014	8	4152868
rs11783438	8	4153438
rs10503249	8	4153834
rs11776902	8	4158386
rs4875330	8	4164349
rs10096058	8	4165916
rs4875334	8	4166391
rs2036547	8	4169124
rs17069952	8	4173962
rs10086105	8	4175588
rs6989090	8	4175751
rs10104209	8	4177153
rs10091031	8	4177588
rs10094349	8	4177720
rs3990909	8	4200701
rs1036556	8	4204712
rs7833644	8	4206941
rs11986849	8	4215192
rs11781210	8	4221514
rs11785556	8	4223569
rs6558873	8	4225651
rs7001908	8	4228333
rs1438211	8	4228348
rs2163303	8	4228462
rs7815034	8	4238427
rs13270991	8	4244607
rs17070159	8	4253714
rs17070162	8	4253847
rs4875102	8	4272100
rs4875356	8	4371279
rs12056740	8	4378785
rs11136749	8	4379107
rs6996668	8	4380342
rs7357469	8	4397922

rs6558891	8	4398160
rs1526335	8	4399459
rs4875112	8	4404404
rs7007873	8	4540722
rs1471419	8	4548162
rs10503270	8	4549341
rs974790	8	4553929
rs10105216	8	4554122
rs2725081	8	4556202
rs1457179	8	4643853
rs17071408	8	4647612
rs4143928	8	4648223
rs7016863	8	4650579
rs17071462	8	4654109
rs1561356	8	4657463
rs17071510	8	4658756
rs4256614	8	4659584
rs4875387	8	4662739
rs11774281	8	4670158
rs10106947	8	4790703
rs6558948	8	4800118
rs12549291	8	4800757
rs11991285	8	4809776
rs12676398	8	4811413
rs814409	8	9970776
rs17151064	8	9990317
rs963592	8	10010714
rs12682291	8	56209626
rs9643832	8	56211316
rs16921239	8	56212279
rs4737900	8	56221035
rs9643838	8	56243233
rs9792156	8	56243326
rs16921354	8	56265394
rs4738044	8	56363676
rs2067776	8	56369847
rs16921563	8	56370015
rs7844897	8	56370375
rs16921571	8	56375314
rs4458852	8	56375684
rs2975985	8	56526641
rs2975982	8	56528005
rs955364	8	56530910
rs955363	8	56531010
rs17364239	8	56606117
rs10504196	8	56609912
rs13251514	8	114001060
rs13262072	8	114002277
rs13270310	8	114024371
rs12681949	8	114024906
rs822337	9	5439154
rs860290	9	5443198

rs822340	9	5443260
rs6651524	9	5448575
rs1411262	9	5449419
rs10114060	9	5451729
rs3780394	9	5454376
rs2297137	9	5455732
rs12352536	9	114968962
rs16911386	9	114980305
rs16911401	9	114980426
rs12236187	9	114994790
rs16932083	9	115085093
rs10981714	9	115088798
rs10981725	9	115103567
rs17156303	10	1458978
rs2805550	10	1468725
SNP_A-1884928	10	1478086
rs7909290	10	14002150
rs7075279	10	14003737
rs1534627	10	14009545
rs17154219	10	14009613
rs11258737	10	14021384
rs7069045	10	14023724
rs2457853	10	14036943
rs2446588	10	14093193
rs11258796	10	14096388
rs7909093	10	14102399
rs10906571	10	14102903
rs11812453	10	14147207
rs7901065	10	14148649
rs11813322	10	14157476
rs11813757	10	14157496
rs7099140	10	14158230
rs11258916	10	14257268
rs17154774	10	14258973
rs7100072	10	14268075
rs11258934	10	14271251
rs7088164	10	14351332
rs1218345	10	14352255
rs17155012	10	14360697
rs1218450	10	14369010
rs1218471	10	14385664
rs1218407	10	14389815
rs1218419	10	14397464
rs1218430	10	14402724
rs1218444	10	14408837
rs1717513	10	14418514
rs11006908	10	28499859
rs2887214	10	28502939
rs2985526	10	28515730
rs7093404	10	55899661
rs11004332	10	55910699
rs9299552	10	55911196

rs7070685	10	67469653
rs10996854	10	67478039
rs10996855	10	67479542
rs10996857	10	67479652
rs10509256	10	67487829
rs7099996	10	67489855
rs7096109	10	67513478
rs10996884	10	67515943
rs10822710	10	67517492
rs10996893	10	67525942
rs10996895	10	67529571
rs7909235	10	68044090
rs10822852	10	68045806
rs9664633	10	68052538
rs7077544	10	68060453
rs10997276	10	68064882
rs4344385	10	68073129
rs4471324	10	68073195
rs4746612	10	68074945
rs4746616	10	68077020
rs2394270	10	68085541
rs1007740	10	68086926
rs6480193	10	68087595
rs2394275	10	68088305
rs2394276	10	68089183
rs12241930	10	68093177
rs10509273	10	68094727
rs2620933	10	68777876
rs10762166	10	68780531
rs9414956	10	68783569
rs16924415	10	68784394
rs12253494	10	77590241
rs11001663	10	77600836
rs12245369	10	77621464
rs2476953	10	105742493
rs1774594	10	105762403
rs805680	10	105773636
rs2483533	10	116302488
SNP_A-2160006	10	116319593
rs3824819	10	116321020
rs3830026	10	118413562
rs9633727	10	118416353
rs12572326	10	118417080
rs2420323	10	118417530
rs11197791	10	118417635
rs17095102	10	118424035
rs2100100	10	118427409
rs17095105	10	118428972
rs12771818	10	118434329
rs2921966	10	118437365
rs1900500	10	118438068
rs1665659	10	118438288

rs3010471	10	118445593
rs3010491	10	118455190
rs10787720	10	118463943
rs735412	10	118469221
rs11197806	10	118471756
rs740599	10	118496745
rs740598	10	118496889
rs2011695	10	118497209
rs740597	10	118498629
rs4372362	10	122634982
rs754869	10	122645708
rs11199636	10	122650763
rs10829893	10	129066258
rs11017876	10	129090954
rs2296631	10	129091178
rs11821431	11	4626790
rs17224476	11	4630364
rs16925767	11	4634929
rs726703	11	12362352
rs16911256	11	12379334
rs11022340	11	12380408
rs10831809	11	12385952
rs10831810	11	12386069
rs1426588	11	12387302
rs4485089	11	12387937
rs11022346	11	12391521
rs4450154	11	12394102
rs7118571	11	12394252
rs16936	11	12423357
rs7930590	11	12426777
rs10831826	11	12427045
rs11022360	11	12435643
rs13451	11	19164417
rs10741763	11	19181294
rs7104244	11	19186454
rs2958194	11	35915081
rs2942400	11	35923417
rs263097	11	35925869
rs12575741	11	35926252
rs11033481	11	36161566
rs4756293	11	36161844
rs11033482	11	36165154
rs7928915	11	36171534
rs16928516	11	36176824
rs16928518	11	36176918
rs11602141	11	36178984
rs7943733	11	36180817
rs12280774	11	36181534
rs11828824	11	36182024
rs870716	11	36182096
rs2422181	11	36182547
rs801741	11	65673660

rs11227418	11	65675617
rs801739	11	65679165
rs1115508	11	65679725
rs6591207	11	65706250
rs580891	11	65711257
rs7941431	11	65716733
rs515184	11	65716987
rs10791855	11	65720313
rs10466673	11	65721958
rs10128616	11	87926085
rs3862362	11	87926577
rs12283602	11	87927999
rs9299908	11	87928204
rs6483397	11	88095026
rs6483399	11	88097946
rs904495	11	88106009
rs2892293	11	88108891
rs1846475	11	88109123
rs6483475	11	88183404
rs10765794	11	88191529
rs11021449	11	88191927
rs12272308	11	88192675
rs12280186	11	88192732
rs12283295	11	88198927
rs12282226	11	88200081
rs11021479	11	88200603
rs11021523	11	88207021
rs560946	11	88323402
rs7110923	11	88324468
rs640541	11	88324615
rs624913	11	88326326
rs1037089	11	105055213
rs11226828	11	105073621
rs632100	11	105094067
rs956753	12	11701825
rs2541129	12	11707392
rs1861484	12	11716839
rs2856312	12	11718922
rs2856320	12	11743762
rs2187641	12	11746557
rs2724588	12	11754082
rs2860431	12	11757330
rs17818498	12	11764555
rs2416883	12	11767661
rs2856325	12	11768057
rs12369491	12	11781775
rs12828066	12	11783232
rs1009951	12	11784163
rs1009953	12	11784395
rs10491989	12	11784622
rs17818624	12	11788113
rs11615175	12	11790225

rs2856338	12	11796019
rs2724602	12	11799123
rs7957338	12	11812572
rs11833885	12	11822254
rs4763725	12	11822422
rs16907264	12	11823130
rs10772506	12	11823325
rs7314811	12	11853840
rs2710293	12	11860837
rs12827379	12	11862890
rs10843167	12	28474060
rs10843169	12	28474251
rs11049606	12	28488413
rs11049615	12	28496352
rs11049630	12	28504440
rs10843178	12	28504701
rs11049633	12	28505448
rs3926041	12	28505976
rs2141203	12	28506128
rs10843179	12	28506325
rs10843180	12	28506421
rs7314502	12	28506500
rs10843181	12	28507024
rs11049656	12	28525905
rs10506037	12	28542413
rs7961961	12	28542871
rs12317472	12	28549152
rs10843189	12	28551185
rs7969086	12	28551473
rs10843192	12	28552956
rs11049684	12	28556626
rs6581336	12	38466665
rs6581338	12	38467601
rs4427625	12	38468873
rs4639999	12	38469949
rs12300180	12	38479702
rs7955109	12	38600637
rs11174338	12	38609908
rs7972838	12	38615024
rs7297783	12	38616188
rs7316296	12	38617431
rs953824	12	38618041
rs11174374	12	38618120
rs11559994	12	38622761
rs6581445	12	38624676
rs2200086	12	38725867
rs11175023	12	38749935
rs11175027	12	38750776
rs10082797	12	38751207
rs11175106	12	38762881
rs10860642	12	99748285
rs11110549	12	99759815

rs11110560	12	99773686
rs2293642	12	104125032
rs17036813	12	104143784
rs11112421	12	104160708
rs11060844	12	129417111
rs2173085	12	129427927
rs11060852	12	129428556
rs880214	13	27096414
rs475916	13	27103077
rs7337722	13	27117158
rs4883960	13	73420062
rs9592955	13	73431915
rs17217378	13	73437717
rs17289662	13	73437964
rs9565064	13	73438417
rs1536027	13	73481792
rs17291339	13	73486524
rs10507821	13	73492535
rs17291465	13	73492660
rs17291723	13	73499936
rs17291799	13	73501916
rs7991281	13	73502080
rs11568637	13	94684855
rs2153673	13	94686824
rs9516546	13	94687679
rs17235124	13	94692042
rs10508021	13	94692121
rs16950829	13	94692386
rs4148442	13	94695759
rs9590213	13	94699826
rs9590224	13	94709749
rs9524870	13	94712655
rs9590227	13	94721126
rs4148427	13	94723675
rs4773867	13	94728621
rs9524879	13	94730011
rs7324283	13	94731671
rs7983813	13	94734859
rs7331135	13	97821530
rs3896398	13	97825401
rs9582224	13	97840159
rs1549836	13	102491230
rs6491729	13	102492286
rs7335602	13	102492514
rs183965	13	102502105
rs279936	13	102502609
rs182195	13	102502829
rs397268	13	102503356
rs1854519	13	102504282
rs279912	13	102504991
rs1886925	13	102505233
rs183963	13	102505285

rs7324197	13	102507261
rs9518912	13	102507473
rs157381	13	102508685
rs9557996	13	102510672
rs9582655	13	102519109
rs1307289	14	54112365
rs1212968	14	54113270
rs1187596	14	54126106
rs17098385	14	60991294
rs3783798	14	60992540
rs17098394	14	60994488
rs10133302	14	60997024
rs8010084	14	60997338
rs11158346	14	60998059
rs912617	14	61003289
rs912618	14	61003344
rs8011286	14	61005927
rs3783786	14	61006977
rs17098433	14	61007336
rs4899049	14	61007908
rs8008798	14	61017755
rs10483736	14	61018970
rs10483737	14	61019403
rs17098514	14	61022592
rs11851966	14	61022609
rs3783769	14	61023040
rs4902064	14	61026577
rs17098535	14	61026984
rs17098542	14	61028043
rs10140070	14	61031278
rs17098569	14	61031660
rs17098610	14	61043310
rs3783759	14	61051517
rs10134093	14	61058766
rs10483742	14	61060072
rs2252351	14	61072304
rs2950274	14	61072425
rs1088669	14	61073480
rs17098753	14	61080169
rs1314922	14	67786720
rs7147214	14	67801071
rs11847821	14	67803505
rs10483809	14	67906122
rs4902574	14	67907178
rs17105494	14	67912383
rs7144111	14	67922063
rs727392	14	68071554
rs1476586	14	68073257
rs2525527	14	68079492
rs17105826	14	68082222
rs963918	14	68131867
rs963917	14	68132076

rs10136316	14	68133274
rs17829311	14	68134319
rs17105940	14	68136312
rs17105965	14	68139347
rs1559538	14	78235361
rs6574465	14	78241305
rs7149617	14	78249224
rs6574467	14	78249498
rs1465481	14	78250799
rs17108796	14	78702575
rs1531634	14	78705764
rs8008994	14	78713021
rs11851562	14	78714963
rs12590183	14	78731932
rs8016735	14	78732379
rs7154580	14	78739528
rs4356397	14	78741293
rs7160828	14	78752560
rs7159606	14	78759089
rs7160122	14	78759192
rs2370937	14	78763320
rs2293836	14	79377043
rs2049826	14	79383063
rs2293839	14	79387213
rs2293847	14	79396835
rs8022988	14	79403317
rs11159422	14	79407737
rs1347891	15	45793870
rs17329501	15	45813308
rs2600867	15	45818522
rs765	15	45822977
rs4775708	15	45824941
rs9888724	15	45826056
rs652281	15	45826380
rs7167021	15	45833273
rs3809485	15	45838748
rs16960030	15	45838944
rs9920019	15	45841022
rs8023364	15	45841364
rs3743276	15	45842411
rs6493294	15	45845090
rs532598	15	45845363
rs624778	15	45861260
rs532707	15	45861363
rs7165320	15	50331716
rs4268699	15	50337054
rs10851510	15	50338189
rs17650635	15	50339192
rs4776033	15	50343964
rs17650694	15	50356256
rs7175663	15	50360167
rs8040562	15	50360812

rs12592927	15	50368775
rs4514621	15	50370445
rs7170506	15	69463289
rs16955547	15	69474191
rs16955551	15	69474538
rs4238652	16	17107166
rs7184743	16	17107812
rs7192758	16	17108938
rs7193152	16	17109163
rs7200466	16	17119046
rs1553088	16	17119483
rs7200513	16	17126976
rs8048747	16	17129729
rs9807009	16	17133351
rs1463460	16	17238380
rs1463461	16	17238450
rs8045551	16	17244992
rs13338458	16	17245007
rs6498067	16	28014873
rs16939814	16	28018715
rs8048659	16	28023752
rs8048569	16	28023902
rs17590103	16	28024594
rs205362	16	28024612
rs13329972	16	28026629
rs11863615	16	28035839
rs189969	16	28038467
rs33044	16	28045467
rs33038	16	28048869
rs245735	16	28055934
rs245732	16	28058480
rs10521144	16	28063021
rs17260630	16	81211536
rs6565051	16	81216229
rs7204454	16	81216695
rs16957898	16	81220939
rs4782722	16	81229666
rs4782726	16	81258834
rs9936363	16	81264849
rs16958140	16	81266662
rs16958145	16	81268319
rs16958148	16	81268357
rs1564029	16	81268370
rs10514554	16	81268866
rs16958275	16	81278207
rs17265361	16	81280441
rs7342783	16	81284387
rs12933997	16	81365790
rs17276565	16	81370055
rs12920400	16	81370460
rs7191150	16	81371443
rs8061163	16	81372531

rs11646849	16	81374752
rs12934135	16	81378367
rs7184633	16	81379514
rs8051326	16	81380213
rs1531436	16	81380827
rs6565065	16	81381053
rs7193817	16	81383662
rs4783277	16	81386902
rs9932137	16	81391528
rs2318184	16	81395462
rs8056951	16	81397804
rs286676	16	81444813
rs16958781	16	81454197
rs16958785	16	81454395
rs4782736	16	81459202
rs4513093	16	81464043
rs7201088	16	81464521
rs16958803	16	81469724
rs17674039	16	81469875
rs11150512	16	81472179
rs2059230	16	81474183
rs918660	16	81477962
rs918661	16	81478238
rs11150513	16	81480895
rs7201829	16	81481182
rs7202226	16	81481390
rs16958826	16	81483050
rs11150518	16	81487229
rs9646331	16	81491933
rs9319578	16	81493544
rs16958840	16	81497693
rs17742591	16	81502215
rs9922544	16	81505974
rs6565089	16	81540534
rs11150524	16	81546433
rs12923390	16	81553706
rs7199767	16	81560851
rs4627328	16	81717335
rs8054536	16	81726200
rs8056019	16	81726277
rs16959371	16	81727918
rs12446981	16	81783353
rs6565151	16	81789528
rs12933683	16	81797607
rs4536460	16	81805289
rs11150550	16	81811260
rs10781996	16	81811817
rs12325628	16	81812818
rs7185989	16	81813459
rs7185229	16	81814269
rs889730	16	81814806
rs10871446	16	81815503

rs8182163	16	81815518
rs1424187	16	81820098
rs1424165	16	81824276
rs1424171	16	81827215
rs11860907	16	81828944
rs1424178	16	81829795
rs9938108	16	81830075
rs1895535	16	81830296
rs2113292	16	81838808
rs889725	16	81845081
rs756458	16	81846617
rs8053104	16	81846984
rs8053119	16	81847020
rs17756425	16	81850840
rs2113294	16	81851629
rs8061579	16	81851914
rs16959994	16	81852852
rs16960036	16	81859526
rs17679500	16	81865050
rs1559439	16	81868424
rs1559440	16	81868770
rs9930051	16	81946193
rs11646728	16	81947943
rs16960303	16	81954238
rs16960306	16	81955608
rs7200009	16	81981765
rs8052043	16	81986485
rs7199636	16	81987403
rs3736239	16	81987702
rs9940088	16	82005733
rs16960421	16	82008129
rs17214677	16	82008463
rs1364309	16	82008540
rs13337525	16	82011695
rs923423	16	82012173
rs9925521	16	82014956
rs16960577	16	82062826
rs17685535	16	82064767
rs931406	16	82066405
rs7498834	16	82067882
rs7206133	16	82319607
rs427585	16	82320567
rs10514596	16	82321641
rs3096277	16	82321705
rs391794	16	82327227
rs435282	16	82335103
rs364960	16	82335277
rs3784990	16	82336571
rs443832	16	82337145
rs12600170	16	82337208
rs16961669	16	82340494
rs1645843	16	82340848

rs3784993	16	82342624
rs2326023	16	82343460
rs418518	16	82344331
rs2326024	16	82350655
rs254340	16	82351597
rs877920	16	82351735
rs9947046	18	369855
rs9964252	18	382795
rs1940439	18	400368
rs9946470	18	32228394
rs9958721	18	32237385
rs8088004	18	32255341
rs1942919	18	55723779
rs4614834	18	55723871
rs4447521	18	55727293
rs4940912	18	55732132
rs10445545	18	55732462
rs2229430	19	7093843
rs2229434	19	7093921
rs2963	19	7114154
rs7259833	19	63171558
rs11670102	19	63171656
rs35826885	19	63178665
rs6135128	20	14290719
rs1475745	20	14300641
rs1998236	20	14301101
rs1998237	20	14301346
rs204651	20	14305685
rs6110516	20	15026343
rs6110518	20	15033205
rs17272483	20	15034442
rs17357440	20	15034862
rs6079664	20	15044768
rs6074844	20	15048284
rs11908639	20	15066513
rs6110527	20	15066761
rs6110528	20	15067097
rs1743471	20	15068323
rs11087123	20	15068744
rs1743473	20	15068767
rs372586	20	15070361
rs6110533	20	15070578
SNP_A-1883579	20	15070605
rs444709	20	15073338
rs1657969	20	15073972
rs17700500	20	15083938
rs808924	20	15084730
rs459322	20	15085117
rs464232	20	15087345
rs459874	20	15087547
rs6034117	20	15092763
rs6043124	20	15102575

rs28696952	20	15102706
rs6043125	20	15109670
rs6079797	20	15315114
rs17374261	20	15320192
rs2327933	20	15327676
rs6079801	20	15329648
rs6079802	20	15330355
rs2021974	20	15330919
rs6110624	20	15409635
rs6043316	20	15410489
rs721318	20	15412529
rs2208135	20	15413513
rs1233738	20	15460552
rs1816000	20	15462094
rs6074914	20	15467613
rs1233727	20	15471985
rs6043354	20	15472717
rs13038859	20	15475445
rs201222	20	15609194
rs6043443	20	15616071
rs16996387	20	15616741
rs6043447	20	15619890
rs7270597	20	15750635
rs12481033	20	15750809
rs6034296	20	15756444
rs16996627	20	15756549
rs7268466	20	15758676
rs16996640	20	15758800
rs2327968	20	15761491
rs12481296	20	15767785
rs4555415	20	15767911
rs6135592	20	15823691
rs717145	20	15826091
rs6514634	20	15827451
rs175781	20	15834294
rs8183396	20	15834612
rs2236008	20	15839419
rs6135598	20	15839973
rs6080021	20	15845768
rs6080024	20	15850299
rs6110831	20	15850454
rs6080026	20	15854219
rs6135608	20	15858218
rs6135612	20	15862579
rs6043629	20	15868298
rs6074969	20	15869963
rs766970	20	15877488
rs6080048	20	15880560
rs8117304	20	31214952
rs17304572	20	31225580
rs8117112	20	31229309
rs2837438	21	40419933

rs2837439	21	40420302
rs448324	21	40429214
rs16999365	21	40434031
rs11911749	21	40446501
rs2837464	21	40451058
rs2837465	21	40453077
rs1016445	21	40453131
rs7280615	21	40454533
rs2205143	21	40459247
rs726104	21	40485110
rs8130768	21	40492714
rs2837499	21	40499689
rs9984703	21	40499746
rs2837500	21	40499765
rs2837508	21	40507618
rs1554940	21	40509697
rs2837510	21	40511147
rs2837512	21	40514545
rs444584	21	40517596
rs415565	21	40517889
rs376508	21	40518055
rs447940	21	40518170
rs2837517	21	40519789
rs435386	21	40520243
rs370432	21	40521016
rs8132437	21	40528196
rs2837525	21	40529361
rs1882758	21	40532666
rs9305695	21	40539677
rs2837534	21	40544164
rs2837535	21	40545262
rs2837536	21	40546492
rs7280961	21	40547818
rs2837552	21	40572993
rs16999614	21	40576908
rs8132571	21	40586166
rs7283487	21	40588759
rs16999652	21	40596365
rs718599	21	40603541
rs2205081	21	40630864
rs9975866	21	40645286
rs8132551	21	40660906
rs2837581	21	40661557
rs2837587	21	40666871
rs2837588	21	40669929
rs16999717	21	40675884
rs741771	21	40728656
rs9967964	21	40732916
rs10439672	21	40735155
rs8129771	21	40735890
rs2837627	21	40737892
rs2837765	21	40948811

rs7279710	21	40957328
rs6517605	21	40957682
rs4818152	21	40958090
rs8127441	21	40960563
rs9977945	21	40962128
rs8134673	21	40970181
rs13047833	21	40978773
rs12628611	22	27983409
rs2857461	22	27998004
rs6006084	22	27998199
rs16992365	22	43266085
rs5764858	22	43266327
rs1962219	22	43266482
rs6007167	22	43266788
rs105248	22	43270063
rs131163	22	43270799
